# Transcriptomic analysis identifies organ-specific metastasis genes and pathways across different primary sites

**DOI:** 10.1186/s12967-020-02696-z

**Published:** 2021-01-07

**Authors:** Lin Zhang, Ming Fan, Francesco Napolitano, Xin Gao, Ying Xu, Lihua Li

**Affiliations:** 1 Institute of Biomedical Engineering and Instrumentation, Hangzhou Dianzi University, Hangzhou, 310000 China; 2 Computer, Electrical and Mathematical Sciences and Engineering Division (CEMSE), Computational Bioscience Research Center (CBRC), King Abdullah University of Science and Technology (KAUST), Thuwal, 23955-6900 Saudi Arabia; 3 Cancer Systems Biology Center, The China-Japan Union Hospital, Jilin University, Changchun, 130033 China; 4 MOE Key Laboratory of Symbolic Computation and Knowledge Engineering, College of Computer Science and Technology, Jilin University, Changchun, 130012 China; 5 Computational Systems Biology Lab, Department of Biochemistry and Molecular Biology and Institute of Bioinformatics, University of Georgia, Athens, GA 30602 USA

**Keywords:** Organ-specific metastasis, Gene expression profiles, Pathway analysis

## Abstract

**Background:**

Metastasis is the most devastating stage of cancer progression and often shows a preference for specific organs.

**Methods:**

To reveal the mechanisms underlying organ-specific metastasis, we systematically analyzed gene expression profiles for three common metastasis sites across all available primary origins. A rank-based method was used to detect differentially expressed genes between metastatic tumor tissues and corresponding control tissues. For each metastasis site, the common differentially expressed genes across all primary origins were identified as organ-specific metastasis genes.

**Results:**

Pathways enriched by these genes reveal an interplay between the molecular characteristics of the cancer cells and those of the target organ. Specifically, the neuroactive ligand-receptor interaction pathway and HIF-1 signaling pathway were found to have prominent roles in adapting to the target organ environment in brain and liver metastases, respectively. Finally, the identified organ-specific metastasis genes and pathways were validated using a primary breast tumor dataset. Survival and cluster analysis showed that organ-specific metastasis genes and pathways tended to be expressed uniquely by a subgroup of patients having metastasis to the target organ, and were associated with the clinical outcome.

**Conclusions:**

Elucidating the genes and pathways underlying organ-specific metastasis may help to identify drug targets and develop treatment strategies to benefit patients.

## Background

Metastasis is a fatal step in cancer progression and is the main cause of cancer-related deaths [1]. Tumor metastasis to different organs is not a random process but is known to show organ-specific preference [2]. Organ-specific metastasis was first described by the “seed and soil” theory proposed by Stephen Paget; according to the theory, certain tumors (the “seeds”) have specific affinity for particular organs (the “soil”) [3]. For instance, colon carcinomas usually metastasize to liver and lung but rarely to bone, brain, and kidneys. In contrast, breast carcinomas, frequently metastasize to most of these organs [4]. Recent discoveries indicate that molecular characteristics of cancer cells and their target tissues cooperate to determine the organ-specific metastasis observed for many tumors, greatly enhancing our understanding of the “seed and soil” theory [5]. Investigation of the mechanisms that mediate site-specific metastasis are likely to lead to the identification of new drug targets for therapy. For instance, targeting the expression of platelet-derived growth factor receptor signaling pathways in tumor-associated endothelial cells and pericytes could inhibit liver metastasis of colon carcinoma [6]. However, the mechanisms of organ-specific metastasis remain an intriguing but unanswered questions in cancer research.

Gene expression profiles are widely used to explore organ-specific metastasis. Several groups have developed microarray-based diagnostic tools to determine a tumor’s site of origin [7, 8] or to predict metastasis sites [9]. For example, the Pathwork Diagnostics® is a well-studied and clinically validated microarray-based gene expression diagnostic test for determining tissue of origin [7, 8]. However, these studies did not elucidate the roles of signatures in the preferences of primary tumor types when spreading to specific distant sites. Several groups have worked on this question, mainly focusing on identifying genes that mediate metastases of one primary cancer type to particular sites [10–12] or whose expression in primary tumors correlates with metastatic recurrence [13]. However, to our knowledge, these markers tended to capture the diversity of primary cancer metastases, especially for breast cancer metastases [10–12, 14]. There has been a lack of systematic research on the commonality of molecular characteristics for different primary tumors metastasizing to the same target organ. Moreover, a well-designed dataset and schema of organ-specific metastasis gene identification are crucial for this type of analysis.

In this study, we investigate organ-specific metastasis by examining gene expression signatures across different tumor types that metastasize to the same organ (e.g., comparing primary breast and lung tumors that both metastasize to the brain). For each metastasis site (brain, liver, or lung), at least two types of primary site were included in the integrated dataset. Then, a rank-based method was used to detect differentially expressed genes (DEGs) between metastatic tumor tissues and the corresponding control tissues. We focused on common DEGs and enriched pathways across all tissues of origin, and investigated these organ-specific metastasis genes and enriched pathways in breast primary tumors. Cluster analysis and survival analysis were used to test whether the organ-specific metastasis genes and pathways were expressed uniquely by a subgroup of patients with metastasis to the target organ, and whether they were associated with clinical outcomes. In conclusion, we present here an analysis to identify signatures that are specific to the common target organ rather than to diverse primary tumor tissue types. The delineation of the roles of these signatures in the interplay between cancer cells and the target organ will lead to a better understanding of organ-specific metastasis and its susceptibilities to treatment.

## Materials and methods

### Datasets

We searched all public databases for transcriptional profiles with clinical data of primary and metastasis site. Nine metastasis microarray gene expression datasets, covering three metastasis sites (brain, liver and lung) [15], were collected from the Gene Expression Omnibus (GEO) database [16]. As shown in Table 1, for each type of metastasis site, at least two types of primary sites were considered. Control tissue samples for each type of metastasis site were also collected from GEO (Table 2).

**Table 1:** Datasets of metastasis samples for three metastasis sites across different primary sites

Primary site	Dataset	#Brain metastasis^a^	#Liver metastasis^b^	#Lung metastasis^c^	Platform	Year
Breast cancer	GSE56493		27		GPL10379	2014
	GSE46141		16		GPL10379	2013
	GSE14020	19	5	18	GPL96, GPL570	2009
	GSE43837	19			GPL1352	2014
	GSE46928	11			GPL96	2013
Colon cancer	GSE41568		79	8	GPL570	2016
	GSE18549		25	6	GPL570	2016
Lung cancer	GSE14108	28			GPL96, GPL570	2010
	GSE18549	6			GPL570	2016
Liver cancer	GSE40367			12	GPL570	2015

^a^Number of brain metastasis samples

^b^Number of liver metastasis samples

^c^Number of lung metastasis samples

**Table 2:** Datasets of control tissue samples for three metastasis sites

Metastasis site	Dataset	#Control^a^	Platform	Year
Brain	GSE7696	4	GPL570	2008
	GSE13162	17	GPL571	2008
	GSE4757	10	GPL570	2006
	GSE35864	6	GPL570	2012
Liver	GSE25097	243	GPL10687	2011
Lung	GSE19804	60	GPL570	2011

^a^Number of brain/liver/lung normal tissue samples

All of nine metastasis microarray gene expression datasets were collected from the Affymetrix platform with relatively consistent quality control. For the two datasets performed on the customized GPL10379 platform (Rosetta/Merck Human RSTA Custom Affymetrix 2.0 microarray), only samples with high quality control (> 50% tumor cell content) were included in the datasets. Additional, among the five datasets metastasized from breast cancer, only one dataset GSE43837 was specific for the HER2+ subtype, this dataset was not excluded from this study in order to enhance the statistical power in DEG detection.

### Dataset integration and DEG detection

To make full use of the information available from multiple datasets, data from different datasets were integrated using the R package virtualArray [17]. The virtualArray software combine data sets of different chip types based on current gene annotations from NCBI database. The integrated expression datasets have their expression values presented as log2-transformed.

Genes that were detected by all platforms (Tables 1 and 2) were kept for the integrated dataset. As non-biological experimental variation or “batch effects” are commonly observed across multiple datasets from microarray experiments [18], conventional expression intensity-based methods such as the significance analysis of microarrays (SAM) were not appropriate here. Instead, as the relative ordering of gene expression within each sample would be rather robust against batch effects and insensitive to data normalization, the rank-based method RankComp was used for DEG identification [19].

RankComp method is based on the relative ordering information of gene expression within each sample. As the relative ordering of gene expression is overall stable for particular types of normal human tissues across common platforms [20], reversal ordering in the disease sample indicate a gene’s up- or down-regulation relative to the other gene for a reversal gene pair. The Fisher’s exact test was used to determine whether a given gene is differentially expressed in a given disease sample by testing the null hypothesis that the numbers of reversal gene pairs supporting its upregulation and downregulation are equal. DEGs at the subpopulation level was identified by using the binomial test to find a non-randomly high percentage of disease samples sharing certain DEGs. The p-values were adjusted by the Benjamini–Hochberg procedure with a 5% false discovery rate (FDR) threshold [21].

### Quantification and statistical analysis of DEG detection

We further estimated effect size for the identified DEGs. To estimate the effect size in unpaired data, expressions of each DEG were assumed to follow normal distributions with different variances in condition *i* and *j* such that $X_{i} \sim N(\mu_{i} ,\sigma_{i}^{2} ), X_{j} \sim \left( {\mu_{j} ,\sigma_{j}^{2} } \right)$. Moreover, the variance between datasets was omitted for the asymptotic estimator. In this more realistic heteroscedastic case, we applied the effect size definition proposed by Kulinskaya [22, 23]:

1 $$\delta_{{{\text{kulinskaya}}}} = \frac{{\mu_{i} - \mu_{j} }}{\sigma }, \sigma = \left\{ {\frac{{q\sigma_{i}^{2} + \left( {1 - q} \right)\sigma_{j}^{2} }}{{q\left( {1 - q} \right)}}} \right\}^{1/2} .$$

Let *n* = *n*_i_ + *n*_j_ and *q* = *n*_j_/*n*, the denominator *σ* could be rewritten as:

2 $$\frac{{ \sigma^{2} }}{n} = \frac{{ \sigma_{i}^{2} }}{{n_{i} }} + \frac{{ \sigma_{j}^{2} }}{{n_{j} }}.$$

This effect size could, therefore, be linked to the Welch t statistic as:

3 $$t_{welch} = \sqrt n \delta_{{{\text{kulinskaya}}}} .$$

A medium effect size threshold of 0.5 was used to further screen the DEGs that were identified by the RankComp method.

In addition, Chi-square test was used to test whether there’s a prominent bias towards available clinical characteristics (such as age and stage) between metastasis tumor and control samples for each dataset in DEG detection.

### Organ-specific metastasis gene identification and function analysis

**Fig. 1:**
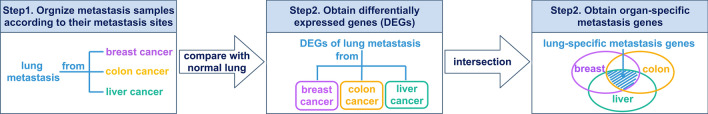
Schema of organ-specific metastasis gene identification. The schema is illustrated by the lung-specific metastasis gene identification

To identify organ-specific metastasis genes, we compared samples grouped according to the metastasis sites. As illustrated in Fig. 1, in the case of lung metastasis, we obtained different sets of DEGs by comparing lung metastasis samples from different tissues of origin with the same group of lung control samples. Then, to exclude housekeeping genes from the resulting gene set, we selected overlapping DEGs in lung metastases from different tissues of origin rather than selecting genes with similar expression across lung metastasis samples from different tissues of origin. These were considered to be lung-specific metastasis genes.

As individual genes often act in concert and may be responsible for multiple effects, organ-specific genes should be considered in a global context. Separate enrichment analysis was performed for up- and down-regulated organ-specific metastasis genes using known biological pathways [24], which were downloaded from Kyoto Encyclopedia of Genes and Genomes (KEGG) [25] and Gene Ontology (GO) database [26] in August, 2019. The hypergeometric distribution model was used to test whether the number of organ-specifics metastasis genes annotated in a functional category was significantly greater than would be expected by random chance [27]. The p-values were adjusted using the Benjamini–Hochberg procedure [21].

## Results

### High percentage overlapping DEGs among primary cancer metastases to the same organ

**Fig. 2:**
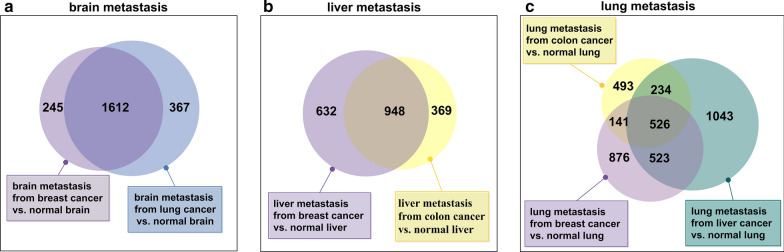
Similarity of DEGs identified in brain, liver, and lung metastases. Proportionate Venn diagram of DEGs are enumerated and labeled in boxes with colors matching the circles in brain (**a**), liver (**b**) and lung metastasis (**c**)

Among the 9797 genes included in the integrated dataset, 1857 DEGs were identified between the 49 metastatic brain samples originating from breast cancer and 37 brain control tissue samples, denoting this list of DEGs as L_bb_. Another 1979 DEGs were identified between 34 metastatic brain samples originating from lung cancer and the same brain control samples, denoted as L_lb_. Between these two lists, 1612 DEGs overlapped, accounting for 87% of L_bb_ (p < 1.0E−06, hypergeometric test) and 81% of L_lb_ (p < 1.0E−06, hypergeometric test). There was a particularly large overlap for brain metastases from breast cancer and lung cancer (as shown in Fig. 2). Similar results were observed for liver and lung metastases (Fig. 2): 948 DEGs overlapped for liver metastases originating from breast and colon cancer, accounting for the 72% of the shorter DEG list (p < 1.0E−06, hypergeometric test); and 526 DEGs were shared for lung metastases from three primary sites, covering one third of the shortest DEG list.

**Fig. 3:**
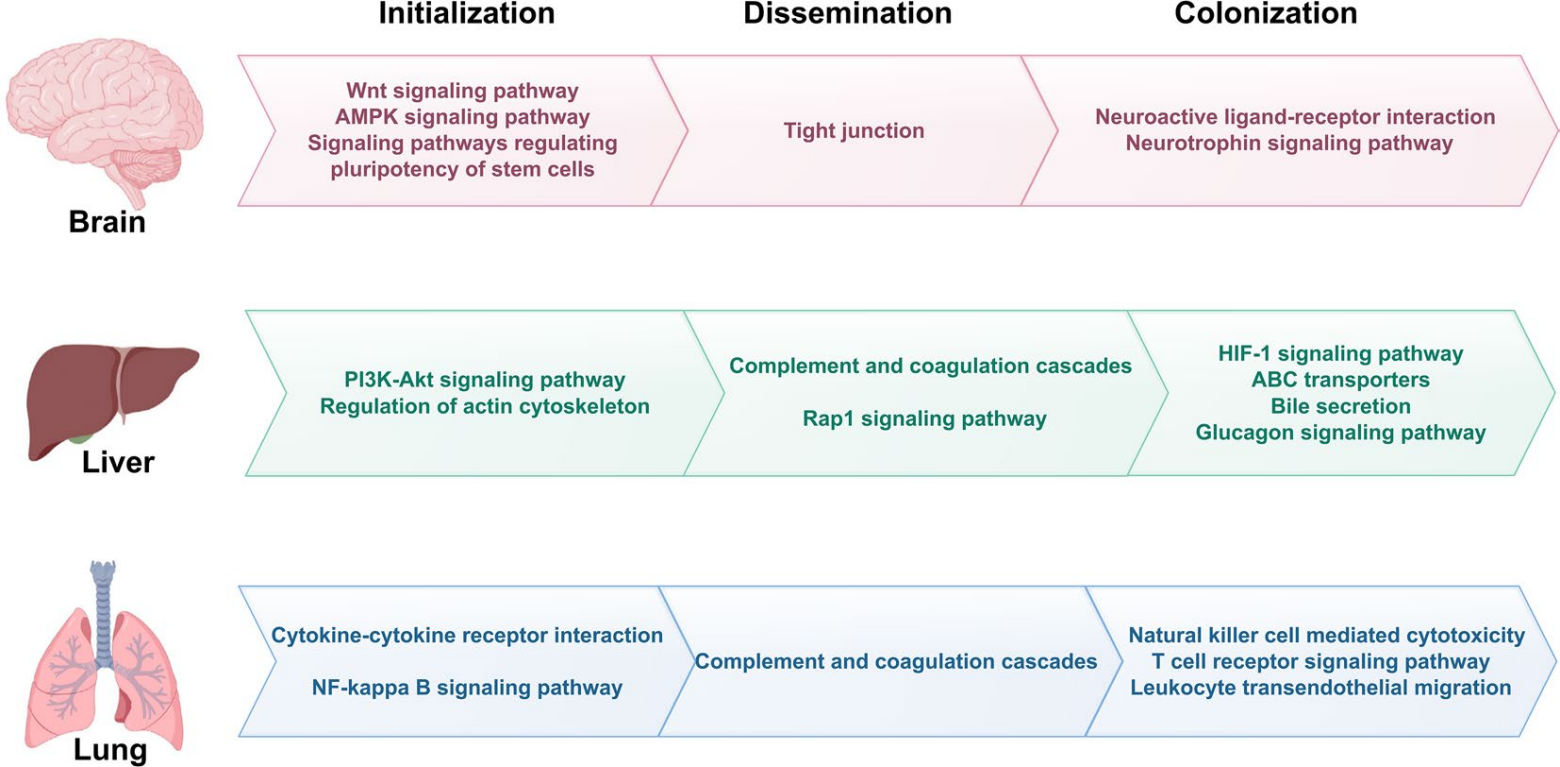
Prominent pathways in the process of metastasis. Some of enriched pathways of common DEGs are highlighted in the initialization, dissemination and colonization stage for brain, liver and lung metastasis

The overlapping DEGs that metastasized from different primary sites to the same metastasis site were identified as organ-specific metastasis genes (brain-specific, liver-specific, and lung-specific metastasis genes are listed in the Additional file 1: Table S1). Using functional analysis, we characterized the prominent molecular events in the initialization, dissemination, and colonization stages of brain, liver, and lung metastasis [28], as illustrated in Fig. 3 and detailed in the following sections.

### Genes and pathways mediating metastasis of different cancers to the brain

We analyzed the 1612 overlapping DEGs between L_bb_ and L_lb_ that both metastasized to the brain tissue, and found that 59% of the common DEGs were down-regulated relative to their levels in brain control tissue. Using functional enrichment analysis with a 5% FDR threshold, 16 KEGG signaling pathways (as shown in Table 3) and 81 fine-grained GO biologic processes (Additional file 1: Tables S2 and S3) were identified that were specific to brain metastasis.

**Table 3:** KEGG pathways enriched for brain metastasis specific genes

DEG	KEGG pathway	FDR^a^	%Genes^b^ (%)	#Genes^c^
Up-regulated	Neuroactive ligand-receptor interaction	< 0.001	12	42
	Cytokine-cytokine receptor interaction	< 0.001	11	32
	PI3K-Akt signaling pathway	0.007	9	31
	JAK-STAT signaling pathway	0.017	10	17
	Signaling pathways regulating pluripotency of stem cells	0.025	10	15
	RIG-I-like receptor signaling pathway	0.043	13	9
Down-regulated	Autophagy	0.001	18	24
	Tight junction	0.003	13	23
	Dopaminergic synapse	0.006	14	19
	AMPK signaling pathway	0.007	15	18
	Endocytosis	0.010	11	28
	Wnt signaling pathway	0.018	13	20
	Inositol phosphate metabolism	0.019	16	12
	FoxO signaling pathway	0.023	13	17
	Lysosome	0.034	13	16
	Neurotrophin signaling pathway	0.039	13	15

^a^The p-values corrected with Benjamini–Hochberg

^b^The percentage of associated genes in each signaling pathway

^c^The number of associated genes in each signaling pathway

Some of these 16 pathways were found to be related to brain-specific dissemination and colonization (Fig. 3), and most of them had not been previously reported. In the initialization stage, pathways regulating pluripotency of stem cells was enriched among the up-regulated brain-specific metastasis genes. Stem cells are pluripotent and proliferate, the long-term tumorigenic potential of some tumors may rely on a small proportion of stem cells endowed with the capacity to indefinitely self-renew [28]. In the dissemination stage, the brain is protected by the blood–brain barrier, which distinguishes it from other organs. Interaction with and penetration of the blood–brain barrier by cancer cells is a key step in colonization to brain tissue. The blood–brain barrier relies on tight junctions between the endothelial cells of the brain capillaries to provide a closed environment for the brain [29]. The tight junction pathway, which was enriched among the down-regulated brain-specific metastasis genes, comprises a number of proteins including occludins, claudins, and junctional adhesion molecules. Claudins are key integral proteins that regulate blood–brain barrier permeability, these proteins not only regulate paracellular permeability but also play a role in the regulation of tight junction assemblies [30]. The canonical Wnt pathway also has a central role in brain angiogenesis and blood–brain barrier formation [29]. In the colonization stage, the neuroactive ligand-receptor interaction pathway was enriched among the up-regulated brain-specific metastasis genes, the GABAR family (GABARAP, GABARAPL1, GABBR1, and GABBR2), the glutamate receptor family (GRM5 and GRIN2A), and the cholinergic receptor family (CHRNA1, CHRNA3, and CHRNA6) showed marked differential expression. Neman et al. [31] showed that breast-to-brain metastatic tissue and cells displayed a GABAergic phenotype similar to that of neuronal cells, which could represent a malignant adaptation required for metastasis to the brain. Glutamate receptors have also been implicated in the pathophysiology of various human malignancies [32]. These results indicate that neuroactive ligand-receptor interactions might also be an important pathway in the development of brain metastases. Finally, significant disturbances were found in pathways such as autophagy and endocytosis, which have crucial roles in metastatic processes. Malignant tumor cells must overcome these various forms of cell death in order to metastasize.

### Genes and pathways mediating metastasis of different cancers to the liver

We analyzed the 948 overlapping DEGs between liver tumor samples metastasized from breast and colon cancer and found that 63% of these DEGs were up-regulated relative to their levels in liver control tissue samples. Using functional enrichment analysis with a 5% FDR threshold, we identified 16 KEGG signaling pathways (as shown in Table 4) and 35 GO biologic processes (Additional file 1: Tables S4 and S5) specific to liver metastasis.

**Table 4:** KEGG pathways enriched for liver metastasis specific genes

DEG	KEGG pathway	FDR^a^	%Genes^b^ (%)	#Genes^c^
Up-regulated	Cell cycle	0.036	10	12
	FoxO signaling pathway	0.047	7	10
	Glucagon signaling pathway	0.048	7	7
Down-regulated	Rap1 signaling pathway	< 0.001	9	19
	Bile secretion	0.001	13	9
	PI3K-Akt signaling pathway	0.001	6	22
	Platelet activation	0.002	10	12
	MAPK signaling pathway	0.004	6	18
	Ras signaling pathway	0.005	6	15
	Focal adhesion	0.008	7	13
	Complement and coagulation cascades	0.009	9	8
	HIF-1 signaling pathway	0.009	8	9
	Regulation of actin cytoskeleton	0.012	6	13
	ErbB signaling pathway	0.020	8	7
	Peroxisome	0.020	8	7
	ABC transporters	0.021	11	5

^a^The p-values corrected with Benjamini–Hochberg

^b^The percentage of associated genes in each signaling pathway

^c^The number of associated genes in each signaling pathway

We identified some pathways involved in liver-specific dissemination and colonization that had not been previously linked to liver-specific metastasis (Fig. 3). In the initialization stage, the significantly enriched reorganization of the actin cytoskeleton pathway reflects the required migratory property [33]. In the dissemination stage, the Rap1 signaling pathway plays an important part in the regulation of endothelial barrier function, a process controlled largely by cell–cell adhesions and their connections to the actin cytoskeleton [34]. In the colonization stage, hepatocellular carcinoma is one of the most hypoxic tumors with median oxygen levels as low as 0.8%. The significantly disturbed hypoxia-inducible factor-1α (HIF-1) signaling pathway is capable of mediating cell–cell communication and has an essential role in inducing metastasis [35, 36]. In addition, it is well established that the immune system is crucial to the micro-metastatic microenvironment. The outgrowth of tumor metastases appears to be linked to inflammation; two significantly enriched pathways, platelet activation and complement and coagulation cascades, are likely to play a role in this. Finally, a large proportion of the enriched pathways were enriched in the metabolism system, including glucagon signaling pathway, bile secretion and ABC transporters. This is probably due to the importance of glucose homeostasis and biliary metabolism in liver microenvironment [37], liver metastases are highly glycolytic and consume local glucose. While the significantly enriched FOXO signaling pathway also plays an important part in the integration of insulin signaling with glucose homeostasis [38]. Various ABC transporters in the liver are key players that safeguard hepatocytes and avoid toxicity due to over-accumulation of bile acid [39]. The combination of an alternative ABC transporter with a novel substrate may prove an effective chemo-preventive or therapeutic strategy.

### Genes and pathways mediating metastasis of different cancers to the lung

Lung is the second most common metastasis site. Tumors of the breast, colon, pancreas, and liver all tend to metastasize to the lung [40]. Using functional enrichment analysis with a 5% FDR threshold, 18 KEGG signaling pathways (as shown in Table 5) and 139 GO biologic processes (Additional file 1: Tables S6 and S7) were identified that are specific to lung metastasis, some pathways involved in lung-specific dissemination and colonization were illustrated in Fig. 3.

**Table 5:** KEGG pathways enriched for lung metastasis specific genes

DEG	KEGG pathway	FDR^a^	%Genes^b^ (%)	#Genes^c^
Up-regulated	JAK-STAT signaling pathway	0.002	7	11
	Cell cycle	0.022	5	6
	Glycolysis/gluconeogenesis	0.032	6	4
Down-regulated	Hematopoietic cell lineage	< 0.001	12	12
	Cytokine-cytokine receptor interaction	< 0.001	7	21
	Toll-like receptor signaling pathway	< 0.001	11	11
	TNF signaling pathway	< 0.001	10	11
	Leukocyte transendothelial migration	0.001	9	10
	NF-kappa B signaling pathway	0.002	9	9
	T cell receptor signaling pathway	0.002	9	9
	Natural killer cell mediated cytotoxicity	0.006	7	9
	B cell receptor signaling pathway	0.006	9	7
	Chemokine signaling pathway	0.006	6	11
	Complement and coagulation cascades	0.006	8	7
	Adherens junction	0.010	8	6
	NOD-like receptor signaling pathway	0.012	6	10
	Th17 cell differentiation	0.016	7	7
	Cell adhesion molecules (CAMs)	0.024	5	8

^a^The p-values corrected with Benjamini–Hochberg

^b^The percentage of associated genes in each signaling pathway

^c^The number of associated genes in each signaling pathway

The broad surface area and numerous capillaries of lung tissue provide opportunities for cancer cells to adhere, extravasate, and colonize [40]. The significantly enriched cell adhesion molecules (CAMs) pathway has an important role in adhesion of cancer cells to the vascular endothelium [41] and can be induced by the significantly enriched JAK-STAT and NF-kappa B signaling pathways in endothelial cell [42]. The endothelial layer in the lung also has tight junctions between endothelial cells and an intact basement membrane; thus, it represents a more restrictive barrier for extravasation compared with bone or liver [2]. The significantly enriched JAK-STAT might be involved in modulating permeability via effects on cell proliferation [43]. Finally, in the colonization stage, immune responses interact with inflammation, angiogenesis, and cancerized stroma, remodeling the microenvironment to favor colonization [44]. The natural killer cell-mediated cytotoxicity pathway is enriched by significantly down-regulated lung metastasis genes, there is a consensus that natural killer cells exert cytotoxicity against metastatic tumor cells [45]. Activation of epithelial–mesenchymal transition in tumor cells during the metastasis cascade is accompanied by altered cell-surface ligands, recognizable by T cell infiltration, which is crucial to tumor microenvironments and has been extensively studied in primary tumors [46]. However, T cell-dependent mechanisms involved in organ-specific metastasis remain underexplored.

### Validation of brain-specific metastasis genes in primary breast tumor

Genes and pathways related to organ-specific metastasis are expected to be both biologically meaningful and clinically relevant. Taking brain-specific metastasis as an example, the corresponding genes and pathways might be expressed uniquely by a subgroup of patients that suffered metastasis to the brain and associated with clinical outcome.

**Fig. 4:**
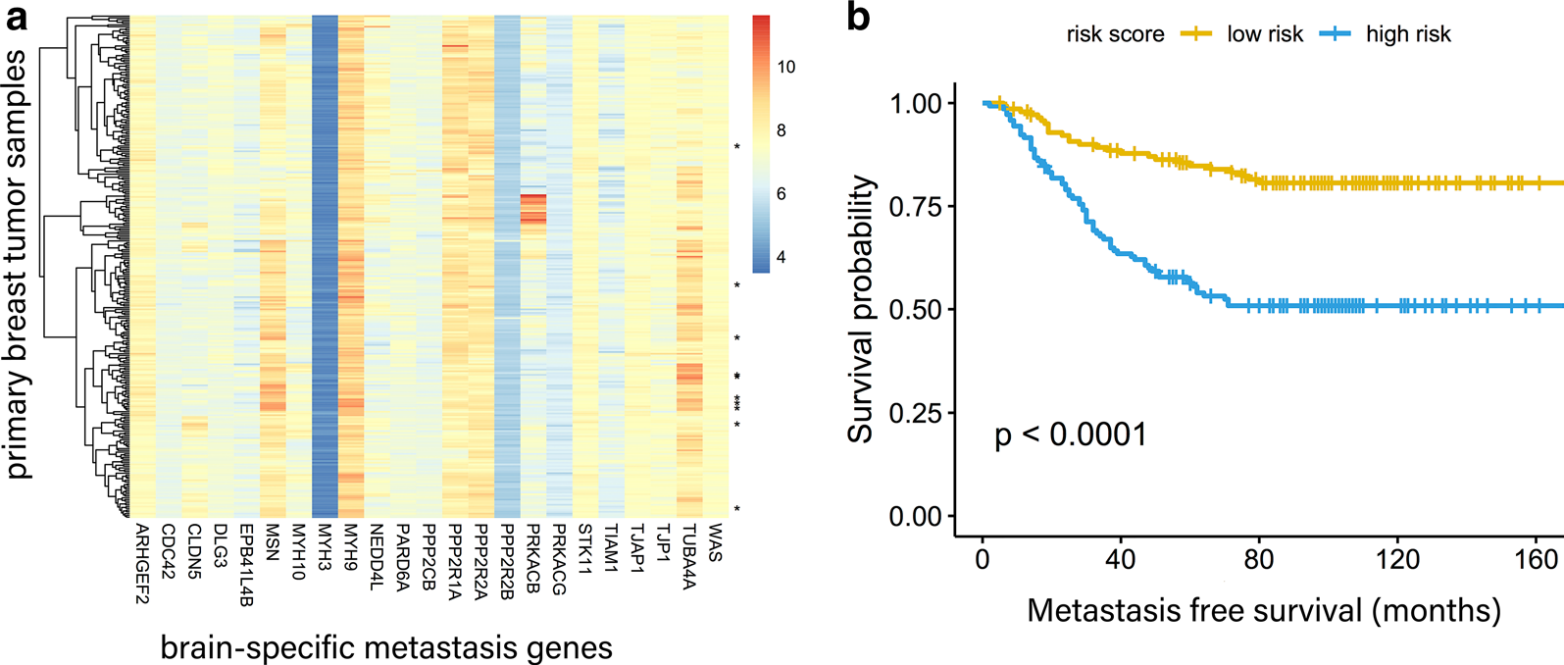
Brain-specific metastasis signature in primary breast tumors. **a** Hierarchical clustering of 268 primary breast cancer patients was performed with 23 genes in the tight junction pathway. A dendrogram of the tumors is shown on the left, tumors from patients who developed brain metastasis were denoted with asterisk marks; **b** metastasis-free survival between low- and high-risk groups of primary breast cancer patients distinguished based on the risk score. The p-value of survival difference by logrank test was shown

To test this, the validation dataset GSE2034 of 286 primary tumors from breast cancer patients was taken from the GEO database. The clinical data of these patients was obtained from a previous report [12], including follow-up observations of metastasis site and metastasis-free survival time. A univariate Cox proportional hazards model was constructed to correlate the expression levels of brain-specific metastasis genes with survival outcomes. Overall, 28% of 1612 brain-specific metastasis genes were significantly associated with metastasis-free survival. For each pathway enriched with respect to the 1612 brain-specific metastasis genes (Table 3), the risk index was calculated as a linear combination of the gene expression values for the brain-specific metastasis genes involved in this pathway, weighted by their estimated regression coefficients in Cox proportional hazards regression modeling [47]. Using the median risk index value as a cut-off point to distinguish high- and low-risk groups, 13 out of 16 pathways showed a significant difference in metastasis-free survival between the two groups (log rank test, p < 0.05). For example, the tight junction pathway distinguished patients at high risk from those at low risk of developing brain metastases (Fig. 4b; log rank test, p < 1.0E−06). Furthermore, the brain-specific metastasis genes involved in the tight junction pathway were used to cluster the dataset hierarchically; most of the brain metastasis patients (marked with asterisks) were clustered together (Fig. 4a). Manual inspection of branches in the dendrogram revealed a group of primary tumors concordantly expressing many elements of these genes, especially high expression of TUBA4A and MSN. These results indicated that a clinically relevant subgroup of patients express certain combinations of brain metastasis signature genes and show differences in metastasis-free survival compared with other patients.

## Discussion

The mechanisms by which different tumor types spread to specific organs, and the identification of the genes and pathways involved, is an emerging topic of investigation in current cancer research. We have systematically analyzed organ-specific metastasis with respect to several organs across all available primary sites. For each metastasis site, we focused on the common DEGs and enriched pathways across all primary sites. This approach has the potential to identify genes that are specific to the target organ rather than to the primary tumor type. Rank-based method RankComp was used for DEG identification, which enabled us to enlarge the sample size and compare samples collected from different cohorts and microarray platforms. However, DEGs were identified only among the genes detected by all platforms; other potential DEGs could have been missed.

We identified a set of genes and pathways that characterized organ-specific metastasis for each metastasis site. Many of the genes identified had not previously been linked to organ-specific metastasis. For example, 53 of the identified brain-specific metastasis genes overlapped with the 243 genes involved in breast cancer metastasis to the brain reported by Bos et al. [12]. The additional genes, including members of the GABAR family, the glutamate receptor family, and the cholinergic receptor family, which were enriched in the neuroactive ligand-receptor interaction pathway, could have important roles in the colonization process in metastasis to the brain. As another example, 15 of the identified lung-specific metastasis genes overlapped with the 95 genes involved in breast cancer metastasis to the lung reported by Minn et al. [11]. The newly identified lung-specific metastasis genes and pathways revealed characteristics at different stages of the lung-specific metastasis process. For example, the CAMs pathway enables cancer cells to attach to the endothelium in the target organ, and the Ig-CAMs (ICAM1, ICAM3) have important roles in this process [41].

The organ-specific metastasis-related pathways identified for each target organ in this study were not necessarily exclusive of each other, possibly owing to the similar characteristics shared by different metastasis organs. For example, the JAK-STAT signaling pathway was enriched in both lung-specific and brain-specific metastasis. This might be due to the functional similarity of the vasculature in the lungs and brain, constituted by a continuous layer of endothelial cells with well-developed tight junctions [48]; whereas the microvasculature in liver is fenestrated. Moreover, tight junction enriched in brain-specific metastases and adherens junction, CAMs signaling pathway enriched in lung-specific metastases provided further evidence that brain and lung metastatic cells need to overcome tight vascular barriers to colonize their target organs. The common pathways also could be induced by multifunctional genes that cooperate with cancer-specific metastasis genes. The pathways enriched by these multifunctional genes reflect general enhancements in the process of metastasis. For example, the PI3K-Akt signaling pathway, which was enriched in both brain and liver metastasis, has multiple roles in regulating survival, cell growth, differentiation, cellular metabolism, and cytoskeletal reorganization of cells in cancer. Moreover, it is noteworthy that, many biology pathways, such as Inositol phosphate metabolism, Wnt signaling pathway, PI3K-Akt signaling pathway, Tight junction, MAPK signaling pathway, TGF-beta signaling pathway and Ras signaling pathway, were strongly associated with cell polarity in the process of metastasis.

Organ-specific metastasis is an intriguing but complex problem. Attempts to understand this phenomenon molecularly have yielded many useful gene markers. However, to our knowledge, these markers tended to explore the diversity of primary cancer metastases, especially for breast cancer metastases. In contrast, our approach more tended to capture the required molecular alterations to colonize and adapt to certain metastasis organs. Many identified genes and pathways are of previously unknown relevance to organ-specific metastasis. Further investigations of the mechanisms that mediate site-specific metastasis are likely to lead to the identification of new targets for therapy.

## Conclusion

In this article, we attempted to explore the commonality of different primary tumors metastasizing to the same organ. For each organ, a substantial proportion of DEGs that metastasized from different primary sites were overlapped. These organ-specific metastasis genes revealed an interplay between the molecular characteristics of the cancer cells and those of the target organ. Specifically, the neuroactive ligand-receptor interaction pathway and HIF-1 signaling pathway were found to have prominent roles in adapting to the target organ environment in brain and liver metastases, respectively. The identified organ-specific metastasis genes and pathways were validated using a primary breast tumor dataset. Survival and cluster analysis showed that organ-specific metastasis genes and pathways tended to be expressed uniquely by a subgroup of patients having metastasis to the target organ, and were associated with the clinical outcome.

## Supplementary Information

**Additional file 1: Table S1.** Brain-, Liver- and Lung-specific metastasis genes compared to corresponding control tissues. **Table S2. **Gene Ontology (GO) biology processes enriched for up-regulated brain-specific metastasis genes. **Table S3. **Gene Ontology (GO) biology processes enriched for down-regulated brain-specific metastasis genes. **Table S4. **Gene Ontology (GO) biology processes enriched for up-regulated liver-specific metastasis genes. **Table S5. **Gene Ontology (GO) biology processes enriched for down-regulated liver-specific metastasis genes. **Table S6. **Gene Ontology (GO) biology processes enriched for up-regulated lung-specific metastasis genes. **Table S7. **Gene Ontology (GO) biology processes enriched for down-regulated lung-specific metastasis genes.

## Data Availability

All data generated or analysed during this study are included in this article and its Additional file.

## Abbreviations

DEG: Differentially expressed genes
FDR: False discovery rate
KEGG: Kyoto Encyclopedia of Genes and Genomes
GO: Gene ontology
GEO: Gene Expression Omnibus
SAM: Significance analysis of microarrays
