# Supplementary material for: Transcriptomic analysis identifies organ-specific metastasis genes and pathways across different primary sites
Source: J Transl Med. 2021 Jan 7;19:31. doi: 10.1186/s12967-020-02696-z (PMC7791985; doi:10.1186/s12967-020-02696-z)
Supplement: Supplementary file 1 — Additional file 1: Table S1. Brain-, Liver- and Lung-specific metastasis genes compared to corresponding control tissues. Table S2. Gene Ontology (GO) biology processes enriched for up-regulated brain-specific metastasis genes. Table S3. Gene Ontology (GO) biology processes enriched for down-regulated brain-specific metastasis genes. Table S4. Gene Ontology (GO) biology processes enriched for up-regulated liver-specific metastasis genes. Table S5. Gene Ontology (GO) biology processes enriched for down-regulated liver-specific metastasis genes. Table S6. Gene Ontology (GO) biology processes enriched for up-regulated lung-specific metastasis genes. Table S7. Gene Ontology (GO) biology processes enriched for down-regulated lung-specific metastasis genes. [file 12967_2020_2696_MOESM1_ESM.docx]

**Table S1.** Brain-, Liver- and Lung-specific metastasis genes compared to corresponding control tissues.

| **Breast-metastasis specific genes (1612)** | A4GNT, ABCD1, ABI1, ACAD8, ACE, ACHE, ACLY, ACTC1, ACTL7A, ADAM10, ADAM30, ADAMDEC1, ADAMTS12, ADAMTS2, ADAMTS20, ADH6, ADIPOQ, ADRB3, AFP, AIPL1, AKAP1, ALX4, AMBN, AMN, ANGPT4, ANGPTL7, ANXA2P3, APOL5, AQP8, ARF4, ARFGEF1, ARID1A, ASB4, ASCC3, ATAD2B, ATF1, ATOH1, ATP1B3, ATP2C2, ATP6AP2, ATXN3L, AZGP1P1, AZU1, B3GALT5, BACH1, BCL2L10, BCL7C, BET1, BMP10, BMP15, BNC2, BRCA2, BRD7P3, BRDT, BRIP1, BRS3, BSPRY, BTG4, C6orf15, C7, C8orf17, CALCR, CAPN11, CAPN7, CASP8, CASZ1, CCDC134, CCDC144A, CCDC81, CCDC88C, CCDC91, CCIN, CCKAR, CCL8, CCNJ, CCNL1, CCR4, CCR8, CCR9, CD164, CD28, CD84, CDH17, CDSN, CDX4, CER1, CFL1, CH25H, CHIA, CHODL, CHRM2, CHRM4, CHRNB3, CKAP4, CLDN1, CLDN16, CLDN17, CLDN18, CLDN3, CLIC3, CLIC5, CLSPN, CMKLR1, CNGA1, CNOT3, CNTLN, COL10A1, COL15A1, COL4A3, COL4A6, COL6A3, COLEC10, CPA2, CPA4, CRHR2, CRISP1, CRLF2, CRYGA, CRYGB, CSF2RB, CSHL1, CSTF1, CTAGE1, CTSE, CXCL6, CYP11B1, CYP27B1, CYP2C19, CYP3A43, DAZL, DCT, DCTN6, DCUN1D1, DDX19A, DEFA5, DEFB126, DEPDC1, DES, DKK4, DLAT, DLK1, DMC1, DMXL2, DNAH3, DNAI2, DSC1, DSC2, DSCR4, DSG2, DSG3, DUSP10, DUSP21, E2F8, EBF2, EFNA4, EGF, EHD4, EHF, EIF3E, ELK3, ELK4, ELSPBP1, EML4, ENG, EPB41L4B, EPHA2, ERCC4, ERCC6L, ERCC8, EREG, ESM1, ESR2, EXOSC2, EXT2, FAM120A, FAM163A, FAM90A1, FANCC, FAT2, FBN1, FBXL4, FBXW4P1, FGF16, FGF4, FGF5, FGFBP1, FLT3, FNBP4, FOXN1, FPGT, FSHB, FSHR, FZD10, FZD6, G6PC2, GABPB1, GABRG3, GAD2, GALR3, GCLM, GDF3, GFRA3, GH1, GH2, GHSR, GIF, GK2, GLI2, GLUD2, GNAT1, GOLGA4, GPR1, GPR12, GPR132, GPR15, GPR45, GPRC5D, GPRIN2, GPX5, GRHL2, GRID2, GRPR, GSG1, GTF2I, GTPBP8, GUCY2C, GUCY2F, GYPB, HAND1, HAVCR1, HBBP1, HDAC9, HEATR3, HELLS, HESX1, HIF3A, HIST1H2AB, HIST1H2AJ, HIST1H2BB, HIST1H2BH, HIST1H3A, HIST1H3G, HIST1H4A, HIST1H4D, HIST1H4G, HIVEP1, HNRNPC, HOXA1, HOXA11, HOXC5, HOXD1, HOXD12, HSD3B1, HSD3B2, HSF1, HSPA6, HSPB7, HTR1A, HTR3A, HTR7, HYAL4, IAPP, IDUA, IFNA10, IFNA16, IFNA2, IFNA4, IFNA5, IFNA8, IFT57, IGHM, IL10, IL15, IL15RA, IL17A, IL17RC, IL22, IMPAD1, INCENP, INF2, INHBC, INPP5A, INPP5B, INTS6, IRF6, ISG20L2, ISL1, ISLR, ITGBL1, JRKL, KCNAB3, KCNK13, KCNK2, KDELR3, KIF14, KIR2DL4, KISS1, KLF10, KLF3, KLHL11, KLHL4, KLK13, KLK5, KRIT1, KRT2, KRT20, KRT33A, KRT36, KRTAP1-1, KRTAP1-3, L2HGDH, LAD1, LAG3, LALBA, LAMB1, LAMB4, LAMP3, LATS1, LBX1, LDHC, LDLR, LGALS14, LGR5, LIM2, LIPF, LMTK2, LONRF3, LOX, LOXL1, LPO, LRCH3, LRTM1, LSR, LY75, LY9, LYZL6, MAGEB2, MAGEB3, MAGEB4, MAGEC1, MAGEC2, MAGEC3, MAGOHB, MALL, MAP2K2, MAP3K6, MAPK13, MATN1, MBTPS2, MC2R, MC3R, MC4R, MCFD2, MCL1, MCTP2, MED23, MEP1A, MEP1B, MFAP2, MIA2, MLLT10, MLN, MMP19, MMP2, MMRN2, MOS, MPZL2, MRPL39, MS4A5, MSH4, MSN, MTFR1, MTHFD2L, MUC4, MUC7, MYBPC2, MYL2, MYO1D, MYOC, MYOZ2, NCR1, NDUFA4L2, NDUFB4, NEIL3, NEK7, NEUROD4, NEUROG1, NFE2L3, NFRKB, NGDN, NID2, NIPSNAP3B, NKX2-1, NMBR, NMU, NODAL, NOL6, NOS2, NPPC, NPVF, NPY2R, NR0B1, NR1H2, NR2C1, NTF3, NTS, NUP50, NUP98, ODF1, OIP5, OPLAH, OPRM1, OR10H3, OR10J1, OR12D3, OR1A1, OR1A2, OR1D2, OR1G1, OR2B2, OR2J2, OR2S2, OR2W1, OR3A1, OR51E2, OR5I1, OR6A2, ORM1, OTUD4, OVOL1, OXT, P2RY2, P2RY4, PACSIN2, PAEP, PAPOLB, PAPPA, PAWR, PAX4, PAX7, PAX9, PBLD, PCDHB1, PCDHB6, PCDHB8, PDC, PDCD1LG2, PDE3A, PDGFC, PDHA2, PDLIM5, PEX13, PFKFB1, PGC, PGK1, PGK2, PHLDA2, PHOX2B, PIGA, PIK3C2A, PITX1, PKP3, PLSCR1, PLXNA3, PMAIP1, PMCH, PMM2, PMS1, PPA2, PPP1R3A, PPP2R3A, PRG2, PRICKLE3, PRKACG, PRKDC, PRL, PRM1, PRND, PRR16, PRRG2, PRRG4, PRSS12, PRSS8, PSG1, PSG3, PSG6, PSTPIP2, PTGER4, PTK7, PTPN14, RBL1, RBMXL2, RBPJL, REG1A, REN, RFXAP, RLN1, RLN2, RNF125, RNF17, RNF186, RPS24, RRAS2, RREB1, RRM1, RSRC1, RUNX2, S100A3, SAV1, SCGB1A1, SCGB1D1, SCN4A, SDAD1, SEC23IP, SEC24A, SECTM1, SERPINB13, SERPINB2, SERPINB5, SERPINB7, SH2D2A, SH3GL3, SH3TC1, SH3YL1, SHB, SIGLEC15, SIGLEC6, SIPA1L3, SIX6, SLC12A2, SLC12A3, SLC22A2, SLC22A8, SLC25A15, SLC25A21, SLC28A2, SLC39A2, SLC45A2, SLC5A4, SLC5A5, SLC5A7, SLC6A2, SLC6A5, SLC9A1, SLCO1A2, SMPX, SNAI2, SNRPE, SNRPF, SNX5, SOX18, SOX21, SPACA1, SPAM1, SPRR1A, SPRR1B, SPTLC1, SRY, SS18, SSX3, STAG1, SUMO1, SVIL, TAAR2, TACR3, TAS2R1, TAS2R13, TAS2R14, TAS2R16, TAS2R3, TBC1D8B, TBR1, TBX4, TCP11, TCP11L1, TDRD12, TEX11, TEX15, TFCP2L1, TGIF1, TIPRL, TLE1, TM4SF20, TMC5, TMEM131, TMEM14B, TMEM53, TMEM62, TMPRSS11D, TNFAIP8, TNFRSF11A, TNFRSF4, TNFSF11, TNFSF15, TNFSF8, TNMD, TNN, TNP2, TOM1L1, TOMM22, TRHR, TRMT5, TRPC2, TRPC3, TRPV5, TSC22D3, TSHB, TSKS, TTC23, TTPA, TUBGCP3, TULP2, UBE2M, UBQLN3, UCKL1, UCP1, UGT2B28, ULBP2, UMOD, UNC93B1, UPK2, USH2A, USP29, UTP18, VARS, VDR, VENTXP1, VSX1, WDHD1, WDR4, WDR73, WDYHV1, WISP1, WNT16, WNT2, WNT6, ZBTB40, ZCCHC8, ZDHHC4, ZIC1, ZIC4, ZNF124, ZNF223, ZNF277, ZNF440, ZNF587, ZNF665, ZNF668, ZNF710, ZNF75D, ZNF93, ZNHIT2, ZP2, ZPBP, ZZEF1, AAK1, AARS, AATK, ABAT, ABCA3, ABCB6, ABCB9, ABCG1, ABHD6, ABR, ACACB, ACADVL, ACAP1, ACBD4, ACOT7, ACTR10, ACTR1A, ACVR1B, ADAR, ADD1, ADD3, AFF3, AFF4, AGAP2, AGT, AGTPBP1, AHCYL1, AHSA1, AHSG, AKR1A1, ALDH9A1, ALDOC, ALKBH4, ANAPC13, ANKFY1, ANKRD11, ANKRD12, ANKRD36B, ANKS1A, ANKS1B, AOAH, AP1G1, AP1G2, AP1S2, AP3D1, APBA2, APBB1, APC, APH1B, APLNR, APLP1, APOBEC3G, APOC3, APOL2, APPBP2, AQP5, AQP9, ARC, ARF3, ARHGEF10, ARHGEF2, ARHGEF6, ARID3B, ARL2, ARL2BP, ARL3, ARL6IP4, ARNT2, ARRB1, ASAP3, ASB13, ASCL1, ASGR2, ASH1L, ASMTL, ASTN1, ATG16L1, ATG2B, ATG3, ATP2B4, ATP2C1, ATP6V0E2, ATP6V1F, ATP6V1G2, ATP6V1H, ATP9A, ATRNL1, ATRX, AUP1, B3GAT1, B9D2, BAALC, BAAT, BACE1, BAG4, BAIAP3, BASP1, BCAP29, BCAS4, BCAT2, BCKDHA, BCL2, BCL6, BCL9, BEND5, BEX4, BIN2, BLMH, BNIP3L, BRD2, BTBD7, BTN3A1, C14orf132, C16orf45, C19orf53, C1orf21, C1orf216, C1QTNF3, C2CD2L, C2orf42, C2orf72, C3AR1, C5orf30, C6orf47, C9orf78, CACNB3, CAMK1D, CAMKK2, CAMKV, CAMLG, CAMP, CAP1, CAP2, CBFA2T3, CBL, CBR1, CBX1, CBX5, CBX7, CCDC130, CCDC25, CCDC92, CCNI, CCNK, CD180, CD22, CD2BP2, CDC14B, CDC16, CDC42EP4, CDK5, CDK5R1, CDK5R2, CDK5RAP2, CDKN2D, CDO1, CDV3, CELSR2, CELSR3, CES2, CFB, CHD7, CHERP, CHGA, CHMP1A, CHMP7, CHN1, CHN2, CHRD, CHSY1, CIAO1, CLCN3, CLCN6, CLDN5, CLIC1, CLPB, CLTB, CNP, CNPY4, CNTNAP1, COASY, COL4A3BP, COPS6, COPS7A, CORO2B, COX6B1, COX7A2, CREBBP, CRIM1, CROCC, CRTAP, CRYBB3, CSAD, CSK, CSNK2B, CSRP1, CTDP1, CTDSP2, CTNNA2, CTNND2, CTSD, CTSZ, CUEDC2, CYFIP1, CYFIP2, CYLD, CYP46A1, CYP4F12, DAAM1, DAAM2, DAB1, DAPK2, DCLK2, DDN, DDX1, DDX17, DDX24, DDX25, DDX41, DDX6, DEF8, DENND1C, DENND4B, DERL2, DIRAS2, DLG4, DNAJA1, DNAJC8, DNASE1L2, DNM1, DNTTIP2, DOCK3, DOK1, DPH2, DPP6, DPYSL2, DPYSL4, DTNA, DUSP26, DYNC1LI2, DYNLL1, E2F4, EBP, ECH1, EDF1, EGR3, EHBP1, EHD2, EI24, EIF1, EIF2S3, EIF3K, EIF4B, ELAC2, ELAVL4, ELMO2, ELP3, ENC1, ENO2, ENOX2, ENTPD2, EPB41L1, EPB42, EPN1, ERCC1, ERGIC3, ETFB, EVI2A, EVL, EXOSC10, EXOSC9, EXTL3, FAAH, FABP1, FADS3, FAIM2, FAM102A, FAM107A, FAM131B, FAM169A, FAM3A, FAM49A, FAM49B, FBRS, FBXL2, FBXO31, FBXO38, FBXO42, FBXW11, FBXW7, FCER2, FEZ2, FGD2, FGFR3, FGL1, FIG4, FKBP10, FKBP1B, FKBP2, FKBP3, FKBP8, FOXO3, FSD1, FSTL3, FTCD, FTO, FURIN, FUT1, FUT7, FXR2, FXYD3, FXYD6, FZD6, GABARAP, GABARAPL1, GABBR1, GABBR2, GADD45G, GAL3ST4, GALNT1, GALT, GAP43, GART, GBF1, GDI1, GDI2, GGA3, GLRX5, GLTP, GMPR, GNAO1, GNB1, GNG3, GNL1, GOLGA2, GPATCH4, GPATCH8, GPM6A, GPM6B, GPR162, GPR37, GPRASP1, GPRC5B, GPSM3, GRAMD1B, GRAP2, GRB2, GRIA1, GRIA3, GRIN1, GRM4, GRWD1, GSK3A, GSK3B, GTPBP2, HADHA, HADHB, HAGH, HAMP, HAPLN2, HARS, HCFC1R1, HDAC11, HDC, HEATR6, HECA, HIPK2, HK2, HNRNPA2B1, HNRNPH1, HNRNPUL2, HPCA, HR, HSD17B14, HSF4, HSPA9, HSPH1, HTT, HUWE1, IDS, IFNAR2, IGF1R, IL16, IL17RB, IMMT, INPP5E, INPP5J, INTS5, IPO8, IQCC, IQSEC1, ITIH1, ITIH3, ITM2B, ITM2C, ITPK1, JAKMIP2, KANK3, KAT2B, KBTBD11, KCNF1, KCNJ2, KCNJ4, KCTD2, KHDRBS1, KHK, KIAA0391, KIAA0408, KIF21B, KIF3A, KIF3B, KIF3C, KIF5C, KIR3DX1, KLHL21, KLKB1, KPNB1, LAGE3, LANCL2, LARS, LCMT1, LCP1, LFNG, LGI1, LGR4, LHPP, LILRA2, LILRA4, LILRB2, LIN37, LMBR1L, LMF1, LMO2, LRRN3, LRRTM2, LSM14A, LST1, LUC7L, LUZP1, MADD, MAGI2, MAL, MAN2A2, MAN2B2, MAP1A, MAP1B, MAP1LC3B, MAP4, MAP9, MAPK11, MAPRE2, MAPT, MAST3, MBP, MC1R, MED27, MED7, MEF2C, MEGF8, MEGF9, METTL1, MFAP3L, MGA, MGAT3, MGLL, MLC1, MLEC, MLLT1, MLLT11, MMP24, MMS19, MOAP1, MOCS1, MPHOSPH10, MPP1, MPP3, MPPED2, MRAS, MRPL16, MRPL41, MRPL52, MSI1, MT1F, MTCH1, MTF1, MTMR4, MTRF1L, MTSS1L, MTUS1, MUC6, MUM1, MXD1, MXI1, MYCBP2, MYH10, MYH3, MYL5, MYO5A, MYOM2, N4BP2L1, NAALADL1, NACAD, NADSYN1, NAGPA, NAP1L3, NCAM1, NCOA1, NCOA4, NDN, NDRG4, NDST1, NDUFB2, NDUFB7, NDUFS6, NECAB2, NEFH, NEFL, NELL2, NFE2L1, NFIX, NGRN, NIF3L1, NIPSNAP1, NLGN3, NLGN4X, NMT1, NOL4, NOLC1, NOP2, NOSIP, NOVA2, NPL, NPR2, NQO2, NR1D2, NR2F6, NRGN, NRN1, NRXN2, NTM, NUAK1, NUBP2, NUCKS1, NUDC, NUDT3, NUP188, NUP214, OGG1, OPCML, OPTN, OSBPL8, OTUD7B, OXA1L, P2RX2, P2RY13, PAAF1, PAF1, PAK1, PAN2, PAQR6, PARD6A, PARP6, PARP8, PBX2, PCBP4, PCDH1, PCDH17, PCDH9, PCM1, PCYT1A, PCYT2, PDE1B, PDE2A, PDE4A, PDIA4, PDPK1, PEBP1, PEG3, PELP1, PEMT, PEX5, PFDN4, PFKFB4, PFKM, PGP, PHF1, PHF3, PHF7, PHKG2, PHYH, PHYHIP, PIGQ, PIK3CD, PIK3R2, PIN1, PINK1, PIP4K2A, PIP5K1C, PIPOX, PITPNA, PJA2, PKNOX1, PKP4, PLA2G5, PLEKHM1, PLEKHO1, PMM1, PMVK, POFUT1, POFUT2, POGK, POLR2G, POMT1, PORCN, POU2F2, POU3F3, PPFIA4, PPM1A, PPM1F, PPM1G, PPP1R7, PPP2CB, PPP2R2A, PPP2R2B, PPP3CA, PPP3CC, PRDM2, PRDX2, PRF1, PRKACB, PRKACG, PRKCSH, PRMT2, PSAP, PSD, PSMD2, PSMD8, PSME1, PSRC1, PTK2B, PTPN1, PTPRA, PURA, QDPR, R3HDM1, R3HDM2, RAB11FIP5, RAB26, RAB28, RAB31, RAB33A, RAB36, RAB3A, RABEP1, RALY, RANBP10, RAP1GAP, RAP1GDS1, RASA3, RASL10A, RBM23, RBM39, RCE1, RELN, RFX1, RFX3, RGL1, RGL2, RHOT2, RIMS3, RIN1, RND1, RND2, RNF10, RNF121, RNF130, RNF170, RNF219, RNF220, RNF40, RNF5, ROBO3, ROM1, RPAP2, RPL18, RPL31, RPL35A, RPL36AL, RPL38, RPN2, RPS28, RSF1, RSL1D1, RSRC2, RTF1, RTN1, RTN2, RTN3, RTN4, RUFY3, S1PR1, SAMD14, SAP18, SART1, SASH3, SCAMP3, SCAPER, SCN1B, SCRN1, SCT, SEC13, SEC31B, SECISBP2, SELL, SEMA3F, SEMA3G, SEMA4D, SEMA6A, SEMA6C, SERGEF, SERINC2, SERPINA3, SEZ6L2, SF1, SFRP5, SFXN3, SGSM2, SGSM3, SH2B1, SH3BGR, SHC2, SHC3, SHPK, SHROOM2, SIGLEC7, SIPA1L1, SIRT3, SIRT5, SLAMF7, SLC11A1, SLC12A5, SLC1A2, SLC1A4, SLC22A17, SLC25A12, SLC25A14, SLC25A23, SLC25A28, SLC25A38, SLC25A4, SLC25A44, SLC2A3, SLC2A6, SLC41A3, SLC44A1, SLC48A1, SLC4A3, SLC7A7, SLC7A8, SLC9A6, SLCO3A1, SLIT1, SLURP1, SMARCD3, SMG5, SMPD1, SNAPC2, SNCA, SNF8, SNPH, SNTA1, SNTG1, SNW1, SNX10, SNX11, SNX24, SOBP, SOCS3, SORBS1, SORL1, SORT1, SOX15, SPAG9, SPATA2, SPATA20, SPATS2, SPCS1, SPOCK2, SPTAN1, SRRM1, SS18L2, SSR2, SSRP1, SST, ST3GAL5, STAT5B, STK11, STK38, STMN2, STMN4, STRADA, STX12, STX3, STYXL1, SUPT5H, SYNGR3, SYNJ2, SYNM, SYT11, TACC1, TAF9B, TAGLN3, TALDO1, TANK, TAOK3, TAPT1, TAZ, TBC1D13, TBC1D9B, TBCB, TBKBP1, TBL3, TCEA2, TCEAL4, TCF25, TCF4, TCF7, TEF, TERF2IP, TESK1, TF, TGM2, TGOLN2, THAP3, THAP4, THOC2, THRA, THYN1, TIAM1, TIMM22, TINF2, TJAP1, TK2, TLN2, TMCC1, TMEM184B, TMEM184C, TMEM222, TMOD2, TMUB2, TNF, TNIP2, TNPO3, TNXB, TOB2, TOMM40, TOP1, TOP3A, TPPP3, TRAK2, TRIAP1, TRIM25, TRIM9, TRNAU1AP, TSC1, TSC2, TSR2, TTYH1, TUBA4A, TXNL4A, TXNRD2, TYROBP, UBN1, UBXN4, UCP3, USF2, USP10, USP11, USP19, USP22, USP33, USP4, UTF1, VAMP1, VAPB, VAT1, VBP1, VPS11, VPS28, VPS45, VPS52, WAS, WDR13, WDR41, WDR48, WDR55, WDR82, WFS1, WIPI2, XPC, XPO7, XPOT, XRCC1, XYLT2, YARS, YEATS2, YLPM1, YY1, YY1AP1, ZBTB33, ZC3H7B, ZDHHC7, ZEB2, ZFR, ZHX3, ZNF174, ZNF189, ZNF202, ZNF212, ZNF286A, ZNF345, ZNF35, ZNF358, ZNF362, ZNF385D, ZNF428, ZNF528, ZNF529, ZNF629, ZNF646, ZNF787, ZNF8, ACSL5, CER1, MSN, TJP1, PPP2R1A, PPP2CB, PARD6A, NEDD4L, EPB41L4B, DLG3, ARHGEF18, CDC42, MYH9, WAS, PRKAG1, PRKACG, CLDN3, CLDN5, FIG4, ITPK1, PLCB2, MTMR1, ISYNA1, JUN, ARHGDIA |
| --- | --- |
| **Liver-specific metastasis genes (948)** | ABCA3, ABCC1, ACE, ACPP, ADAM12, ADAM15, ADAM7, ADAT1, ADCY2, ADO, ADRA2C, AFAP1, AGPAT1, AIPL1, ALDH2, ALOX15, AMELY, ANAPC2, ANKLE2, ANKRD2, ANXA6, AP1S1, APEX2, APTX, AQP2, ARHGDIG, ARID3B, ARL6IP4, ASF1A, ASRGL1, ASXL2, ATAD5, ATM, ATP4B, B3GALT5, B4GALT6, B9D2, BAHD1, BANP, BATF, BBS7, BCKDHA, BCORL1, BDNF, BRCA2, BRD1, BRIP1, BTC, C1orf216, C2orf68, C3orf52, C9orf40, CACNA1D, CACNA1E, CACNA1I, CACNG1, CAMSAP1, CAPN11, CARD10, CBLC, CBX4, CBX7, CC2D1A, CCDC70, CCL1, CCNE2, CCNF, CCNG2, CCNO, CCR9, CCS, CCT4, CD70, CDC7, CDH20, CDKN2B, CDS1, CEACAM4, CEBPE, CENPB, CENPI, CEP55, CFD, CHAC1, CHAF1A, CHEK1, CHRNA5, CLEC5A, CLN5, CLN8, CLPP, CLU, CNNM4, CNOT7, COL19A1, CRABP2, CRBN, CREB1, CREB3L1, CRHR1, CROCC, CRTC1, CRYBA4, CSNK1G1, CSTB, CUBN, CUL3, CYCS, CYTH2, DAGLA, DAZAP2, DBF4, DCLK1, DCLRE1B, DCLRE1C, DCUN1D2, DDRGK1, DDX4, DDX54, DDX6, DEPDC1, DGKD, DGKE, DGKQ, DHX40, DIAPH3, DMWD, DMXL2, DNAJC17, DNASE1, DOT1L, DSC1, DSCC1, DUSP21, ECHDC1, EDN3, EFR3B, EGF, EIF3B, ENY2, EP300, EPHA5, EPS15L1, ERCC6L, ERI3, EXOG, FAM160B2, FANCI, FASTK, FBXL4, FBXL8, FBXO5, FCGBP, FGF16, FGF22, FIG4, FKBPL, FKRP, FKTN, FZD3, GAA, GAB1, GABRA2, GABRB1, GAL3ST4, GALR1, GALR3, GBF1, GDAP1, GEMIN8, GFOD2, GFRA4, GGA1, GJB5, GLG1, GLP1R, GNB1L, GP9, GPATCH3, GPKOW, GPR12, GPR143, GPR153, GRB2, GRHL2, GRIN2B, GRK6, GRN, GSK3A, GSTM3, GTF2H5, GTPBP8, GUCA1A, GUCY2D, GUK1, GYS1, HAS1, HCFC1R1, HCN2, HCN4, HEATR3, HIF1AN, HIPK2, HMBOX1, HNRNPUL2, HOXB8, HOXB9, HOXC4, HOXD4, HRK, HS1BP3, HSF1, HSF2BP, HTR2A, HUNK, IFNA10, IFNB1, IFT140, IFT81, IGF1R, IKBKE, IL13, IL17B, IL25, IL3, IL3RA, IMPA2, INF2, ING3, INHA, INPP4B, INPP5K, INSL4, INTS1, IQCH, ISG20L2, ISL1, JAK1, KANK1, KATNA1, KCNA10, KCNC2, KCND1, KCNK10, KCNK7, KCNQ4, KCTD13, KCTD20, KIAA0754, KIF11, KIF14, KIF20A, KIF23, KIF4A, KLHL25, KRIT1, KRT12, KRT2, KRT81, LAS1L, LEF1, LGALS14, LHX1, LIMA1, LIN7B, LMNA, LMNB1, LOXL2, LPAR1, LPAR2, LRP3, LRRC8B, LRRC8E, LTB, LUZP2, LY6H, MAD2L1, MAFG, MAP3K11, MAP3K3, MAP7D1, MAPK8IP1, MAPKBP1, MAPRE3, MBTPS2, MCM10, MCM4, MCPH1, MDM4, MECP2, MED6, MEOX1, MFSD6, MGAT5, MIP, MKI67, MLH1, MLH3, MMP16, MMP17, MMP20, MOCS3, MOG, MPRIP, MRPL39, MRPS22, MSRB2, MSX2, MT4, MTNR1B, MYBPC1, MYEF2, MYH15, MYH8, N4BP2L2, NAIP, NCOR2, NDOR1, NDRG4, NDST2, NDUFB1, NDUFB4, NDUFB6, NDUFC1, NEBL, NEIL3, NEK2, NENF, NES, NEUROG1, NGDN, NKX3-2, NLRX1, NMB, NME3, NPEPL1, NPHS1, NPY2R, NR1D2, NR2C2, NRL, NSMAF, NUP155, NUP88, OCLM, ODF1, OLIG2, OPRM1, OSBPL7, OTOR, OTUD3, OTUD7B, OVOL1, PACSIN2, PALM, PAPOLG, PAQR6, PARP2, PAX2, PBK, PCBP4, PCDHB1, PCYT1B, PDGFB, PDK3, PGGT1B, PGK2, PHF10, PHF14, PHF20L1, PHOX2A, PI15, PIAS4, PITPNM3, PITX1, PITX2, PIWIL1, PKDREJ, PLAU, PLCD1, PLEKHM1, PLK4, PLXNB2, PNMA3, PORCN, POU2F2, PPP3R1, PRDM12, PRICKLE3, PRKDC, PRMT3, PROP1, PRPS1L1, PRR5, PTGER3, PXMP4, QSER1, RAB11FIP4, RAB23, RAB27B, RAB3D, RABGGTA, RACGAP1, RAD50, RAD51AP1, RAF1, RBBP5, RBL1, RBM12B, RCAN3, REEP2, REL, RET, RFC2, RFWD3, RFXAP, RGS17, RHBDD3, RHOT2, RIMS2, RIT1, RLN1, RLN2, RNF19B, RNF216, RNF32, RNFT2, RNPEPL1, ROS1, RPH3A, RPL10L, RPRD2, RPS24, RPUSD2, RRAGC, RSAD2, RSBN1, RUVBL1, S1PR4, SAMD4B, SCAMP1, SCG2, SCGB1A1, SCGN, SCN5A, SEC14L5, SELPLG, SEMA7A, SEMG1, SENP2, SETDB1, SF3A1, SF3B1, SH2D3A, SHCBP1, SIDT1, SIVA1, SKIL, SLA, SLC22A4, SLC25A40, SLC2A11, SLC39A8, SLC41A3, SLC4A8, SLC6A3, SLC7A6, SLC9A2, SLITRK5, SMARCA5, SMC2, SMC4, SMOX, SMYD5, SNAP91, SNAPC2, SNTA1, SOX12, SP1, SP3, SPAG1, SPAST, SPC25, SPHK2, SPN, SPR, SPTLC2, SREBF1, SREBF2, SS18, SSX2IP, STAM2, STIL, STK17B, STMN3, STRN, SULT2B1, SUV39H2, SYT1, SYTL2, TAAR5, TACC3, TAF13, TAF1A, TAF1C, TAF6L, TAS2R14, TBC1D10B, TBC1D13, TBC1D9B, TDP1, TDRD3, TFAP2C, TFDP3, TGFBR1, TGOLN2, THAP10, THOC6, TK1, TLE3, TMC7, TMEM121, TMEM132A, TMEM184B, TMLHE, TMPRSS3, TNFSF18, TNMD, TNNT2, TNP1, TNRC6B, TPCN1, TPO, TRABD, TRADD, TRDMT1, TRIOBP, TRIP13, TRMT5, TROAP, TROVE2, TTC28, TTK, TTLL1, UAP1L1, UBAC1, UBOX5, UCP1, UEVLD, ULBP1, UNC13A, UNC5B, UPK1B, USH1C, USP16, USP36, USP46, USP49, VANGL1, VKORC1, VPREB1, VTI1B, WDHD1, WDR47, WDR76, WNT6, WSCD2, XRCC4, YEATS4, YIF1A, ZBTB32, ZBTB38, ZBTB39, ZCWPW1, ZDHHC4, ZFP37, ZKSCAN5, ZMAT5, ZMIZ2, ZNF136, ZNF14, ZNF148, ZNF174, ZNF184, ZNF225, ZNF24, ZNF282, ZNF318, ZNF354A, ZNF43, ZNF432, ZNF446, ZNF518A, ZNF551, ZNF571, ZNF592, ZNF629, ZNF652, ZNF706, ZNF721, ABHD6, ACSL1, ADAMTS3, AHNAK, AIF1, AIFM1, AKR1B1, AKR1C1, AKR7A3, ANGPT2, ANKFY1, ANKRD40, ARHGAP1, ARHGEF10L, ASGR1, ATG16L1, ATP1A1, ATP6V1G1, AUTS2, AVPI1, BACE1, BAG5, BASP1, BDH2, BMP2K, BRD8, C18orf25, C1orf54, C1QA, C2orf49, C5AR1, C7, C9orf3, CAMK1, CAMK2B, CAPZA1, CASP2, CAV1, CCDC102B, CCL8, CCNK, CCNL1, CD163, CD28, CD79B, CDC14A, CDK2, CFH, CFP, CHD2, CHST7, CLCN4, CLCN6, CLEC1A, CNP, COMT, COX7A1, CREB3L2, CROT, CRYM, CSF1R, CSGALNACT2, CSRP2, CST3, CXCL12, DAAM2, DAB1, DDAH1, DENND4A, DENND5B, DGCR2, DHRS12, DHRS3, DIRAS2, DNAJB9, DOCK5, DSE, DUSP1, ECHDC2, EGFR, EHD4, EIF3E, EIF4E2, ELAVL1, ELK1, ELK4, ENPP3, ESRRA, ETFB, EVL, EXOSC10, F10, F2RL2, FAM118A, FAM168B, FBLN1, FCN3, FKBP11, FKBP2, FNDC3B, FOXN3, FSCN1, FST, GABPA, GART, GATC, GFM1, GJA4, GLE1, GLRX, GNA13, GPR65, GPX3, GRIK1, GRK5, GSN, HADHB, HAGH, HECA, HERPUD1, HEY1, HGD, HHLA3, HIC1, HIPK1, HMOX1, HNRNPD, HSD11B1, HUWE1, IFNAR2, IFT74, IGFBP4, IL11RA, IL1R2, IL4R, IPO8, IREB2, IRS4, ITFG1, ITGAL, KCNIP1, KCNJ4, KHDRBS3, KHK, KIF1C, KLF9, LAMA4, LRRC8D, MAN2C1, MAOA, MAP4K5, MAPK14, MAT1A, MBD2, MBTD1, MDM2, MEF2A, METTL7A, MEX3C, MKLN1, MKNK1, MLLT1, MMD, MMP24, MON2, MPDZ, MPP1, MT1F, MT1X, MTAP, MTHFD1, MTMR10, MTMR12, MTRF1, MYH11, MYH4, MYLK, NDUFA6, NDUFS1, NEDD4, NINJ1, NOL10, NR1H3, NR3C1, NRAP, NRG2, NSDHL, NUDT7, ONECUT1, OPTN, PAFAH2, PAIP2B, PANX1, PAOX, PC, PCBD1, PCF11, PCOLCE, PCSK2, PDE10A, PERP, PHC2, PHC3, PHF7, PHTF2, PIK3CG, PINK1, PIP4K2A, PLCG2, PLEKHA2, PLEKHA4, PLSCR4, POLDIP2, POLR2D, POLR2F, POMP, PON2, POP4, PPARA, PPP1R12A, PPP1R12B, PRELP, PSMC4, PTPRK, PXMP2, RAB21, RALGPS2, RASGRP1, RBM26, RBM47, REPIN1, RGS5, RNF103, RNF113A, RPS6KB1, RRAGB, SCARF1, SEC24D, SEMA4D, SENP6, SERPINB9, SF1, SGK1, SHPK, SLC25A17, SLC25A20, SLC25A44, SLC38A10, SLC7A1, SLC7A2, SLCO2B1, SMAD4, SMG7, SMURF1, SMURF2, SNAP23, SNRK, SORD, SPARCL1, SPATA7, SPCS3, SPG21, SPRED2, SPTBN1, STC1, STEAP3, STX12, SYT11, TAF7L, TAP2, TAPT1, TBC1D15, TCF20, TCF4, TEK, TGDS, TGFA, TGM2, THYN1, TIMP2, TLE1, TMEM100, TMEM115, TMEM156, TMEM176B, TMEM204, TMEM208, TMEM70, TNFAIP2, TNFRSF10D, TNFRSF9, TOPORS, TPM1, TPM2, TPP2, TRPC1, TRPV2, TSC22D2, TSPAN4, TTC33, TUBGCP3, TWIST1, TXLNA, TXNL4A, UBA5, UBASH3A, UBE2H, UBXN1, USH2A, USO1, USP9X, VSIG4, VWF, WASL, WDR43, WDR59, WIPI2, WISP2, WSB1, XPO7, XYLB, YTHDC2, ZBED1, ZDHHC17, ZNF529, ZNHIT1, ABCA1, ABCC2, ABCC9, ABCG5, BAAT, RXRA, SLC10A1, SLC4A4, SULT2A1, UGT2B4, APBB1IP, FLT4, HGF, LCP2, MRAS, NGFR, PDGFA, PDGFRA, PRKD1, RAPGEF2, RAPGEF5, TLN1 |
| **Lung-specific metastasis genes (526)** | ADAM18，ADAMTS6，AFM，AK5，AKR1C4，ANXA10，AQP8，ATP10B，ATP7B，AURKB，BCORL1，BRD8，BRIP1，BUB1，C8A，CADPS，CDC25C，CDKL3，CLCN5，CLSPN，CLTC，COL2A1，COX10，CPN2，CRISP1，CRP，CYP11B1，DEPDC1，DKK4，DUSP21，ELAVL2，ERGIC3，FETUB，GALR1，GIP，GPR45，GRIA2，HELLS，HIST1H2AK，HIST1H3E，HNF4A，HNF4G，IL19，INHBE，KIF20B，KLK6，KRT12，LARP7，LGI1，MAGEL2，MCM10，MELK，MKRN3，MYH8，MYO3A，NANOG，NAT2，NEIL3，NEK2，NSUN3，OGDHL，ONECUT2，OPCML，OR10H3，PDE11A，PGK2，PIK3R4，PITX1，PKDREJ，PLAC1，PMS1，POLQ，PPP1R12B，PPP1R14D，SLC13A3，SLC26A3，SLC2A4，SLC6A15，SORD，SPRR1B，SYT13，TAS2R10，TAS2R14，TCERG1，TDRD1，TEX11，TJP2，TMED3，TRH，TRIM66，TSHB，TTPA，ULBP1，VPS13B，ZNF286A，ZNF770，AGMAT，CCDC144A，CCNF，CENPO，DNAJC24，GPR22，KRAS，MSI1，NAT8B，NEB，PTPRH，RSRC1，SATB2，SCN8A，SF1，SORBS2，SPC25，TPX2，TRIM31，TRPV6，TTK，UBE2C，ACP6，ADH6，AFF2，ALDOB，AZGP1P1，BCL2L14，BHMT，CABYR，EHMT1，EXO1，GNG4，GYPA，HAO1，HIST3H2A，HJURP，HSD3B1，HTR1D，IFNA10，IL21，KCNA10，LAMB4，LEFTY1，MAGEA10，MC5R，MS4A5，NAP1L4，NEUROD1，OR1G1，OR2B6，OR5I1，OR7A5，PDE6H，PEX26，PNLIPRP1，PRDM13，PROC，RECQL5，SLC25A15，SLC8A2，SMPX，SSX3，TLK1，TTTY15，ZNF3，BUB1B，CCNB1，CDC6，CENPI，HMMR，KIF11，KIF14，MAGEB2，MYBPC1，NCAPG2，NUP43，OIP5，PLK4，SERPINC1，SULT2A1，TFG，ARHGEF7，BCAN，CALB1，COL11A1，CTNNA2，KCNJ13，KCNV1，LSM14B，MAGEC1，MMP3，PARD6B，PCDHB13，PRLR，SLC2A1，TAS2R16，THSD4，GRIK1，POF1B，PPFIA4，SLC17A2，ARHGEF12，CCL27，CSHL1，CYLC1，DLX6，GLP1R，IKZF2，NPY，PKN2，PSG3，PTPN2，RALGPS1，SMR3A，TAS2R13，TDRD12，ZPBP，CD3EAP，ENO3，FABP7，KIF2C，ABCA3，ADRB1，ADRB2，ALPL，ARID5A，BANK1，BCL6，BIRC3，C1orf54，CASKIN2，CASP4，CCL11，CD300C，CD33，CD37，CD40，CEBPD，CFP，CLCN4，CLEC10A，CLIC2，CNTN6，COL13A1，CR1，CSF2RA，CSF3，CST7，DENND3，DENND5A，DOK3，DPEP2，DPT，DUSP22，EDEM1，EFNB1，FAM107A，FAM53C，FGL2，FLI1，FOLR3，FUZ，GIT2，GJA4，GMFG，GNA15，GPR20，GPR4，HAS1，HSPB3，ICAM1，IGLL1，IK，IL10RA，IL18R1，IL18RAP，IL4R，INPP5K，ITIH5，JOSD1，JUND，LILRA2，LST1，LYVE1，MAL，MANBA，MAP7D1，MMP28，MNDA，MT1M，NFKB1，NPR1，NR3C1，NUBP1，P2RY14，PDPN，PLEKHO1，PPP1R15A，PPP1R7，PSTPIP1，PXN，RAB8B，RALGDS，RASGRP2，RBMS1，RFX1，RHOBTB2，RIPK1，RNF19B，RUNX3，SGCG，SIPA1，SOX17，SPI1，STARD8，STEAP4，STX12，SWAP70，THBD，TLR2，TOX，TRIM44，UROD，WISP2，ZBTB16，AES，ARHGAP10，ARID1A，BTK，C7，CD69，CLPP，CTNNA1，CYBRD1，CYTL1，DNASE2B，EGFL7，EPOR，FIS1，HK3，IL1R1，ITPKC，LIMS2，MAFF，MMP19，NME5，OLFM1，PEF1，PLEKHM2，PLLP，PSMB10，RASSF9，RBM42，RETN，ROR1，RRAD，SAMSN1，SLC27A3，SNX1，SPAG7，TEKT2，TICAM1，TJP1，VAV1，VPS13D，ACVRL1，AIF1，ARRB1，AVPI1，BCL2A1，CCDC69，CD247，CD55，CRTAM，DCTN6，DENND2A，DHRS9，EFEMP1，EHD2，EMP3，ETF1，FHOD1，FPR1，FPR2，FYN，GIMAP4，GIMAP6，GPR183，GPR65，GUCY2D，GZMH，HYAL2，ICOS，IFNAR2，IKZF3，IL6，IL7R，ISG20，ITK，JAM2，KANK3，KLRF1，LCP1，LILRB5，METAP2，NOP10，PCDH12，PGM5，PIK3CD，PLAUR，PRKCH，PTAFR，PTK2B，PTPN6，RBMS2，RPS6KA1，RSAD2，S100A12，S100A3，S1PR1，SEMA3G，SERTAD2，SLC31A2，SLIT3，STAP1，STK10，STX11，TACC1，TINF2，TNFRSF1B，TNP1，TREM1，VIPR1，WAS，ZNF426，ALDH3B1，ARC，BATF，BMP5，CD163，CD86，CDKL2，CFD，CH25H，CHRDL1，CLDN5，CLEC4A，CREB1，CSGALNACT2，CYTIP，DAAM2，ELK3，ELL2，F8，FABP4，FBXO38，FSTL3，GNLY，GPSM3，GYPC，HIGD1B，IL27RA，JUNB，KLF4，LAMB2，LTBP4，MAP3K3，MCTP1，MT1E，MYOZ1，OS9，PJA2，PNPLA6，PPP1R8，PRPF8，PTGER4，PTPRN2，RAMP2，REL，RPS6KA2，SASH1，SEC14L1，SLC15A2，ST8SIA1，TLR7，TRPV2，VAMP2，VAT1，VPS37B，FBXL5，KIF17，LIFR，RAB11FIP2，TNFRSF10C，CA3，CRISP2，FLII，FTCD，RGS2，SLC43A3，TLR3，ZNF134，ACPP，APOL3，ARID3B，CD160，CSF2，CYFIP2，ICAM3，MAPRE2，PTPRM，RTN1，SH3BP5，SLC12A6，STOM，TBKBP1，UPP1，BMP2，CDC42EP2，FADS3，FBP1，FURIN，FZD10，GABARAPL1，NEDD4L，PAPSS2，RARA，TIAM1，TIPARP |

**Table S2.** Gene Ontology (GO) biology processes enriched for up-regulated brain-specific metastasis genes.

| **GO ID** | **Description** | **FDR ^1^** | **#Genes ^2^** |
| --- | --- | --- | --- |
| GO:0008544 | epidermis development | <0.001 | 40 |
| GO:0048608 | reproductive structure development | <0.001 | 38 |
| GO:0044706 | multi-multicellular organism process | 0.009 | 21 |
| GO:0002521 | leukocyte differentiation | 0.011 | 35 |
| GO:0033141 | positive regulation of peptidyl-serine phosphorylation of STAT protein | 0.015 | 6 |
| GO:1904892 | regulation of STAT cascade | 0.022 | 16 |
| GO:0098742 | cell-cell adhesion via plasma-membrane adhesion molecules | 0.022 | 21 |
| GO:0050673 | epithelial cell proliferation | 0.039 | 29 |
| GO:0030101 | natural killer cell activation | 0.039 | 10 |

^1^ The p-values corrected with Benjamini-Hochberg; ^2^ The number of associated genes in each signaling pathway.

**Table S3.** Gene Ontology (GO) biology processes enriched for down-regulated brain-specific metastasis genes.

| **GO ID** | **Description** | **FDR ^1^** | **#Genes ^2^** |
| --- | --- | --- | --- |
| GO:0007409 | axonogenesis | <0.001 | 59 |
| GO:0010975 | regulation of neuron projection development | <0.001 | 50 |
| GO:0048588 | developmental cell growth | <0.001 | 30 |
| GO:0006914 | autophagy | <0.001 | 51 |
| GO:0061919 | process utilizing autophagic mechanism | <0.001 | 51 |
| GO:0031346 | positive regulation of cell projection organization | <0.001 | 41 |
| GO:0016311 | dephosphorylation | <0.001 | 49 |
| GO:0016049 | cell growth | 0.001 | 50 |
| GO:0006470 | protein dephosphorylation | 0.001 | 36 |
| GO:0031110 | regulation of microtubule polymerization or depolymerization | 0.001 | 15 |
| GO:0007265 | Ras protein signal transduction | 0.001 | 48 |
| GO:0010769 | regulation of cell morphogenesis involved in differentiation | 0.001 | 33 |
| GO:0051656 | establishment of organelle localization | 0.001 | 46 |
| GO:0050808 | synapse organization | 0.001 | 35 |
| GO:0008361 | regulation of cell size | 0.002 | 23 |
| GO:1903829 | positive regulation of cellular protein localization | 0.003 | 37 |
| GO:0031109 | microtubule polymerization or depolymerization | 0.003 | 17 |
| GO:0000422 | autophagy of mitochondrion | 0.003 | 15 |
| GO:0061726 | mitochondrion disassembly | 0.003 | 15 |
| GO:0010506 | regulation of autophagy | 0.003 | 34 |
| GO:0045927 | positive regulation of growth | 0.004 | 30 |
| GO:0035303 | regulation of dephosphorylation | 0.004 | 25 |
| GO:0001558 | regulation of cell growth | 0.004 | 40 |
| GO:0032869 | cellular response to insulin stimulus | 0.004 | 25 |
| GO:0032868 | response to insulin | 0.005 | 29 |
| GO:0098693 | regulation of synaptic vesicle cycle | 0.006 | 10 |
| GO:0060560 | developmental growth involved in morphogenesis | 0.006 | 26 |
| GO:0021801 | cerebral cortex radial glia guided migration | 0.006 | 7 |
| GO:0022030 | telencephalon glial cell migration | 0.006 | 7 |
| GO:0021548 | pons development | 0.007 | 5 |
| GO:1902115 | regulation of organelle assembly | 0.008 | 21 |
| GO:0006892 | post-Golgi vesicle-mediated transport | 0.008 | 15 |
| GO:0031032 | actomyosin structure organization | 0.008 | 23 |
| GO:0019318 | hexose metabolic process | 0.011 | 26 |
| GO:0043523 | regulation of neuron apoptotic process | 0.012 | 23 |
| GO:0046323 | glucose import | 0.012 | 13 |
| GO:2000310 | regulation of NMDA receptor activity | 0.013 | 6 |
| GO:0099173 | postsynapse organization | 0.014 | 15 |
| GO:0008637 | apoptotic mitochondrial changes | 0.014 | 17 |
| GO:0010821 | regulation of mitochondrion organization | 0.015 | 25 |
| GO:0036465 | synaptic vesicle recycling | 0.016 | 10 |
| GO:0043547 | positive regulation of GTPase activity | 0.016 | 38 |
| GO:0048639 | positive regulation of developmental growth | 0.018 | 21 |
| GO:0048857 | neural nucleus development | 0.018 | 11 |
| GO:0048167 | regulation of synaptic plasticity | 0.020 | 20 |
| GO:0035418 | protein localization to synapse | 0.020 | 8 |
| GO:0060627 | regulation of vesicle-mediated transport | 0.020 | 43 |
| GO:0007006 | mitochondrial membrane organization | 0.020 | 17 |
| GO:0051648 | vesicle localization | 0.020 | 27 |
| GO:0032886 | regulation of microtubule-based process | 0.023 | 22 |
| GO:1903146 | regulation of autophagy of mitochondrion | 0.025 | 9 |
| GO:0032386 | regulation of intracellular transport | 0.026 | 36 |
| GO:0046902 | regulation of mitochondrial membrane permeability | 0.027 | 12 |
| GO:0035304 | regulation of protein dephosphorylation | 0.030 | 16 |
| GO:0051235 | maintenance of location | 0.031 | 30 |
| GO:0070997 | neuron death | 0.033 | 30 |
| GO:0000045 | autophagosome assembly | 0.034 | 13 |
| GO:0060078 | regulation of postsynaptic membrane potential | 0.034 | 16 |
| GO:0007158 | neuron cell-cell adhesion | 0.035 | 5 |
| GO:0043087 | regulation of GTPase activity | 0.037 | 41 |
| GO:0050890 | cognition | 0.037 | 27 |
| GO:0099111 | microtubule-based transport | 0.037 | 17 |
| GO:0099177 | regulation of trans-synaptic signaling | 0.038 | 31 |
| GO:0062012 | regulation of small molecule metabolic process | 0.039 | 41 |
| GO:0106027 | neuron projection organization | 0.039 | 11 |
| GO:0044282 | small molecule catabolic process | 0.039 | 37 |
| GO:0019216 | regulation of lipid metabolic process | 0.039 | 33 |
| GO:1905037 | autophagosome organization | 0.039 | 13 |
| GO:1904659 | glucose transmembrane transport | 0.039 | 14 |
| GO:0099504 | synaptic vesicle cycle | 0.041 | 16 |
| GO:0031647 | regulation of protein stability | 0.041 | 25 |
| GO:0120032 | regulation of plasma membrane bounded cell projection assembly | 0.041 | 18 |

^1^ The p-values corrected with Benjamini-Hochberg; ^2^ The number of associated genes in each signaling pathway.

**Table S4.** Gene Ontology (GO) biology processes enriched for up-regulated liver-specific metastasis genes.

| **GO ID** | **Description** | **FDR ^1^** | **#Genes ^2^** |
| --- | --- | --- | --- |
| GO:0000280 | nuclear division | 0.002 | 34 |
| GO:0048285 | organelle fission | 0.002 | 36 |
| GO:0140014 | mitotic nuclear division | 0.004 | 25 |
| GO:0048568 | embryonic organ development | 0.013 | 32 |
| GO:0045787 | positive regulation of cell cycle | 0.033 | 28 |
| GO:0007088 | regulation of mitotic nuclear division | 0.040 | 16 |
| GO:0046834 | lipid phosphorylation | 0.040 | 13 |
| GO:0051054 | positive regulation of DNA metabolic process | 0.040 | 19 |
| GO:0051783 | regulation of nuclear division | 0.040 | 17 |
| GO:0006260 | DNA replication | 0.040 | 23 |

^1^ The p-values corrected with Benjamini-Hochberg; ^2^ The number of associated genes in each signaling pathway.

**Table S5.** Gene Ontology (GO) biology processes enriched for down-regulated liver-specific metastasis genes.

| **GO ID** | **Description** | **FDR ^1^** | **#Genes ^2^** |
| --- | --- | --- | --- |
| GO:0048015 | phosphatidylinositol-mediated signaling | 0.001 | 15 |
| GO:0048017 | inositol lipid-mediated signaling | 0.001 | 15 |
| GO:0072329 | monocarboxylic acid catabolic process | 0.006 | 12 |
| GO:0030258 | lipid modification | 0.013 | 17 |
| GO:0071384 | cellular response to corticosteroid stimulus | 0.013 | 8 |
| GO:0034599 | cellular response to oxidative stress | 0.014 | 17 |
| GO:0030335 | positive regulation of cell migration | 0.021 | 23 |
| GO:0043627 | response to estrogen | 0.022 | 8 |
| GO:0003197 | endocardial cushion development | 0.022 | 6 |
| GO:0006979 | response to oxidative stress | 0.023 | 21 |
| GO:0006957 | complement activation, alternative pathway | 0.025 | 4 |
| GO:0000302 | response to reactive oxygen species | 0.025 | 14 |
| GO:0044282 | small molecule catabolic process | 0.030 | 20 |
| GO:1901654 | response to ketone | 0.030 | 12 |
| GO:1901655 | cellular response to ketone | 0.032 | 8 |
| GO:0015721 | bile acid and bile salt transport | 0.032 | 5 |
| GO:0002448 | mast cell mediated immunity | 0.032 | 6 |
| GO:0032970 | regulation of actin filament-based process | 0.040 | 18 |
| GO:0007566 | embryo implantation | 0.040 | 6 |
| GO:0050663 | cytokine secretion | 0.042 | 12 |
| GO:0006907 | pinocytosis | 0.043 | 4 |
| GO:0048660 | regulation of smooth muscle cell proliferation | 0.043 | 10 |
| GO:0051260 | protein homooligomerization | 0.043 | 16 |
| GO:0031330 | negative regulation of cellular catabolic process | 0.046 | 13 |
| GO:0048659 | smooth muscle cell proliferation | 0.046 | 10 |

^1^ The p-values corrected with Benjamini-Hochberg; ^2^ The number of associated genes in each signaling pathway.

**Table S6.** Gene Ontology (GO) biology processes enriched for up-regulated lung-specific metastasis genes.

| **GO ID** | **Description** | **FDR ^1^** | **#Genes ^2^** |
| --- | --- | --- | --- |
| GO:0007059 | chromosome segregation | <0.001 | 20 |
| GO:0000819 | sister chromatid segregation | 0.001 | 14 |
| GO:0051983 | regulation of chromosome segregation | 0.001 | 9 |
| GO:0140014 | mitotic nuclear division | 0.001 | 14 |
| GO:0032467 | positive regulation of cytokinesis | 0.001 | 6 |
| GO:0000280 | nuclear division | 0.002 | 17 |
| GO:0048285 | organelle fission | 0.002 | 18 |
| GO:0030071 | regulation of mitotic metaphase/anaphase transition | 0.004 | 6 |
| GO:0007088 | regulation of mitotic nuclear division | 0.004 | 10 |
| GO:1902099 | regulation of metaphase/anaphase transition of cell cycle | 0.004 | 6 |
| GO:0007052 | mitotic spindle organization | 0.006 | 8 |
| GO:1905818 | regulation of chromosome separation | 0.006 | 6 |
| GO:0031055 | chromatin remodeling at centromere | 0.016 | 5 |
| GO:0000086 | G2/M transition of mitotic cell cycle | 0.016 | 11 |
| GO:0034508 | centromere complex assembly | 0.029 | 5 |
| GO:0007094 | mitotic spindle assembly checkpoint | 0.029 | 4 |
| GO:0071174 | mitotic spindle checkpoint | 0.029 | 4 |
| GO:1901990 | regulation of mitotic cell cycle phase transition | 0.029 | 14 |
| GO:0009135 | purine nucleoside diphosphate metabolic process | 0.032 | 7 |
| GO:0009179 | purine ribonucleoside diphosphate metabolic process | 0.032 | 7 |
| GO:0071103 | DNA conformation change | 0.037 | 11 |
| GO:1905819 | negative regulation of chromosome separation | 0.040 | 4 |

^1^ The p-values corrected with Benjamini-Hochberg; ^2^ The number of associated genes in each signaling pathway.

**Table S7.** Gene Ontology (GO) biology processes enriched for down-regulated lung-specific metastasis genes.

| **GO ID** | **Description** | **FDR ^1^** | **#Genes ^2^** |
| --- | --- | --- | --- |
| GO:0002703 | regulation of leukocyte mediated immunity | <0.001 | 18 |
| GO:0032496 | response to lipopolysaccharide | <0.001 | 24 |
| GO:0042119 | neutrophil activation | <0.001 | 30 |
| GO:0050900 | leukocyte migration | <0.001 | 29 |
| GO:0002521 | leukocyte differentiation | <0.001 | 29 |
| GO:0051249 | regulation of lymphocyte activation | <0.001 | 28 |
| GO:0002283 | neutrophil activation involved in immune response | <0.001 | 29 |
| GO:0002446 | neutrophil mediated immunity | <0.001 | 29 |
| GO:0071216 | cellular response to biotic stimulus | <0.001 | 18 |
| GO:0043312 | neutrophil degranulation | <0.001 | 28 |
| GO:0002449 | lymphocyte mediated immunity | <0.001 | 22 |
| GO:0042110 | T cell activation | <0.001 | 27 |
| GO:0022409 | positive regulation of cell-cell adhesion | <0.001 | 19 |
| GO:0031349 | positive regulation of defense response | <0.001 | 26 |
| GO:0042092 | type 2 immune response | <0.001 | 8 |
| GO:0002819 | regulation of adaptive immune response | <0.001 | 13 |
| GO:0002460 | adaptive immune response based on somatic recombination of immune receptors built from immunoglobulin superfamily domains | <0.001 | 20 |
| GO:0032103 | positive regulation of response to external stimulus | <0.001 | 19 |
| GO:0001819 | positive regulation of cytokine production | <0.001 | 23 |
| GO:0042113 | B cell activation | <0.001 | 18 |
| GO:0051017 | actin filament bundle assembly | <0.001 | 13 |
| GO:0045088 | regulation of innate immune response | <0.001 | 23 |
| GO:0007159 | leukocyte cell-cell adhesion | <0.001 | 20 |
| GO:0002685 | regulation of leukocyte migration | <0.001 | 14 |
| GO:0050727 | regulation of inflammatory response | <0.001 | 22 |
| GO:0002768 | immune response-regulating cell surface receptor signaling pathway | <0.001 | 24 |
| GO:0050870 | positive regulation of T cell activation | <0.001 | 15 |
| GO:0032635 | interleukin-6 production | <0.001 | 11 |
| GO:0016049 | cell growth | <0.001 | 23 |
| GO:0032944 | regulation of mononuclear cell proliferation | <0.001 | 14 |
| GO:0032943 | mononuclear cell proliferation | 0.001 | 16 |
| GO:0061900 | glial cell activation | 0.001 | 6 |
| GO:0060759 | regulation of response to cytokine stimulus | 0.001 | 13 |
| GO:0050878 | regulation of body fluid levels | 0.001 | 23 |
| GO:0030168 | platelet activation | 0.001 | 12 |
| GO:0045628 | regulation of T-helper 2 cell differentiation | 0.001 | 4 |
| GO:0032970 | regulation of actin filament-based process | 0.001 | 19 |
| GO:0002440 | production of molecular mediator of immune response | 0.001 | 14 |
| GO:0051092 | positive regulation of NF-kappaB transcription factor activity | 0.001 | 11 |
| GO:0110053 | regulation of actin filament organization | 0.001 | 15 |
| GO:0007249 | I-kappaB kinase/NF-kappaB signaling | 0.001 | 15 |
| GO:0090066 | regulation of anatomical structure size | 0.001 | 22 |
| GO:0001558 | regulation of cell growth | 0.002 | 19 |
| GO:0006957 | complement activation, alternative pathway | 0.002 | 4 |
| GO:0045926 | negative regulation of growth | 0.003 | 14 |
| GO:0043405 | regulation of MAP kinase activity | 0.003 | 17 |
| GO:0018108 | peptidyl-tyrosine phosphorylation | 0.003 | 18 |
| GO:0050817 | coagulation | 0.003 | 17 |
| GO:0071356 | cellular response to tumor necrosis factor | 0.004 | 15 |
| GO:1902105 | regulation of leukocyte differentiation | 0.004 | 14 |
| GO:1902905 | positive regulation of supramolecular fiber organization | 0.004 | 12 |
| GO:0032535 | regulation of cellular component size | 0.004 | 17 |
| GO:0001909 | leukocyte mediated cytotoxicity | 0.004 | 8 |
| GO:0001906 | cell killing | 0.004 | 10 |
| GO:0007204 | positive regulation of cytosolic calcium ion concentration | 0.004 | 15 |
| GO:0001959 | regulation of cytokine-mediated signaling pathway | 0.005 | 11 |
| GO:0050920 | regulation of chemotaxis | 0.005 | 12 |
| GO:0031341 | regulation of cell killing | 0.005 | 7 |
| GO:0042035 | regulation of cytokine biosynthetic process | 0.005 | 8 |
| GO:0043547 | positive regulation of GTPase activity | 0.005 | 18 |
| GO:0043087 | regulation of GTPase activity | 0.006 | 20 |
| GO:0034612 | response to tumor necrosis factor | 0.006 | 15 |
| GO:0035690 | cellular response to drug | 0.006 | 16 |
| GO:0030212 | hyaluronan metabolic process | 0.007 | 5 |
| GO:2000116 | regulation of cysteine-type endopeptidase activity | 0.008 | 13 |
| GO:0014013 | regulation of gliogenesis | 0.009 | 8 |
| GO:0042089 | cytokine biosynthetic process | 0.009 | 8 |
| GO:0051607 | defense response to virus | 0.009 | 12 |
| GO:0008360 | regulation of cell shape | 0.010 | 10 |
| GO:0006968 | cellular defense response | 0.010 | 6 |
| GO:0072507 | divalent inorganic cation homeostasis | 0.010 | 19 |
| GO:0032353 | negative regulation of hormone biosynthetic process | 0.010 | 3 |
| GO:2000117 | negative regulation of cysteine-type endopeptidase activity | 0.011 | 8 |
| GO:0045190 | isotype switching | 0.012 | 5 |
| GO:0045429 | positive regulation of nitric oxide biosynthetic process | 0.012 | 5 |
| GO:1904407 | positive regulation of nitric oxide metabolic process | 0.012 | 5 |
| GO:0032351 | negative regulation of hormone metabolic process | 0.013 | 3 |
| GO:0051271 | negative regulation of cellular component movement | 0.013 | 15 |
| GO:0110020 | regulation of actomyosin structure organization | 0.013 | 7 |
| GO:0009612 | response to mechanical stimulus | 0.015 | 11 |
| GO:0045444 | fat cell differentiation | 0.015 | 11 |
| GO:1904645 | response to amyloid-beta | 0.016 | 4 |
| GO:0040013 | negative regulation of locomotion | 0.016 | 15 |
| GO:0003158 | endothelium development | 0.018 | 8 |
| GO:0035810 | positive regulation of urine volume | 0.018 | 3 |
| GO:0008585 | female gonad development | 0.018 | 7 |
| GO:0051591 | response to cAMP | 0.019 | 7 |
| GO:0071248 | cellular response to metal ion | 0.020 | 9 |
| GO:0002831 | regulation of response to biotic stimulus | 0.021 | 8 |
| GO:0046209 | nitric oxide metabolic process | 0.021 | 6 |
| GO:0030838 | positive regulation of actin filament polymerization | 0.022 | 7 |
| GO:0097305 | response to alcohol | 0.022 | 10 |
| GO:0042063 | gliogenesis | 0.023 | 12 |
| GO:0032273 | positive regulation of protein polymerization | 0.023 | 8 |
| GO:0010038 | response to metal ion | 0.024 | 14 |
| GO:2001057 | reactive nitrogen species metabolic process | 0.024 | 6 |
| GO:0001501 | skeletal system development | 0.026 | 18 |
| GO:0045446 | endothelial cell differentiation | 0.027 | 7 |
| GO:0071559 | response to transforming growth factor beta | 0.028 | 11 |
| GO:0090313 | regulation of protein targeting to membrane | 0.029 | 4 |
| GO:0010744 | positive regulation of macrophage derived foam cell differentiation | 0.029 | 3 |
| GO:0007548 | sex differentiation | 0.031 | 12 |
| GO:0051099 | positive regulation of binding | 0.031 | 9 |
| GO:0009615 | response to virus | 0.031 | 13 |
| GO:0005976 | polysaccharide metabolic process | 0.033 | 7 |
| GO:0051384 | response to glucocorticoid | 0.035 | 8 |
| GO:0070266 | necroptotic process | 0.036 | 4 |
| GO:0042267 | natural killer cell mediated cytotoxicity | 0.036 | 5 |
| GO:0045732 | positive regulation of protein catabolic process | 0.038 | 10 |
| GO:0043297 | apical junction assembly | 0.038 | 5 |
| GO:0097178 | ruffle assembly | 0.041 | 4 |
| GO:0046677 | response to antibiotic | 0.041 | 13 |
| GO:0060560 | developmental growth involved in morphogenesis | 0.043 | 10 |
| GO:1990138 | neuron projection extension | 0.045 | 8 |
| GO:0042698 | ovulation cycle | 0.045 | 5 |
| GO:0051346 | negative regulation of hydrolase activity | 0.049 | 16 |
| GO:0036120 | cellular response to platelet-derived growth factor stimulus | 0.049 | 3 |
